# Oxidative stress: fundamentals and advances in quantification techniques

**DOI:** 10.3389/fchem.2024.1470458

**Published:** 2024-10-07

**Authors:** Hari Krishnan Krishnamurthy, Michelle Pereira, Imbaasree Rajavelu, Vasanth Jayaraman, Karthik Krishna, Tianhao Wang, Kang Bei, John J. Rajasekaran

**Affiliations:** ^1^ Vibrant Sciences LLC., Santa Clara, CA, United States; ^2^ Vibrant America LLC., Santa Clara, CA, United States

**Keywords:** oxidative stress, reactive oxygen species, reactive nitrogen species, free radicals, antioxidants, lipid peroxidation

## Abstract

Oxidative species, generated endogenously via metabolism or from exogenous sources, play crucial roles in the body. At low levels, these species support immune functions by participating in phagocytosis. They also aid in cellular signaling and contribute to vasomodulation. However, when the levels of oxidative species exceed the body’s antioxidant capacity to neutralize them, oxidative stress occurs. This stress can damage cellular macromolecules such as lipids, DNA, RNA, and proteins, driving the pathogenesis of diseases and aging through the progressive deterioration of physiological functions and cellular structures. Therefore, the body’s ability to manage oxidative stress and maintain it at optimal levels is essential for overall health. Understanding the fundamentals of oxidative stress, along with its reliable quantification, can enable consistency and comparability in clinical practice across various diseases. While direct quantification of oxidant species in the body would be ideal for assessing oxidative stress, it is not feasible due to their high reactivity, short half-life, and the challenges of quantification using conventional techniques. Alternatively, quantifying lipid peroxidation, damage products of nucleic acids and proteins, as well as endogenous and exogenous antioxidants, serves as appropriate markers for indicating the degree of oxidative stress in the body. Along with the conventional oxidative stress markers, this review also discusses the role of novel markers, focusing on their biological samples and detection techniques. Effective quantification of oxidative stress may enhance the understanding of this phenomenon, aiding in the maintenance of cellular integrity, prevention of age-associated diseases, and promotion of longevity.

## Introduction

“Oxidative stress” is a term that was first coined in 1985 by the German physician, Helmut Sies as an imbalance between the production of oxidants and antioxidant defenses that may result in damage to biological systems (Forman and Zhang, 2021). Since then, the phenomenon has been extensively studied, as it has been implicated in a wide range of diseases, including cancer, neurological disorders, atherosclerosis, hypertension, ischemia, diabetes, acute respiratory distress syndrome, idiopathic pulmonary fibrosis, chronic obstructive pulmonary disease, and asthma (Birben et al., 2012). Reactive oxygen species (ROS) and reactive nitrogen species (RNS) are the key players contributing to oxidative stress generated intrinsically from normal cellular metabolism, and extrinsically, from environmental factors such as toxins, UV radiation, or cigarette smoke (Forman and Zhang, 2021).

Additionally, biological processes such as oxidative phosphorylation, activation of several transcriptional factors, apoptosis, immunity, cell differentiation, and amino acid synthesis produce ROS and RNS (Pizzino et al., 2017; Di Meo et al., 2016). ROS and RNS can be divided into two groups: free radicals and nonradicals. The molecules that contain one or more unpaired electrons contributing to their reactivity are called “free radicals.” On the other hand, when two free radicals share their unpaired electrons, then “nonradical forms” are created (Birben et al., 2012). The ROS that are physiologically relevant include superoxide anion radicals (O_2_^•^ˉ), hydrogen peroxide (H_2_O_2_), hydroxyl radicals (^•^OH), and singlet oxygen (^1^O_2_), which are generally present in cells at low levels (Pizzino et al., 2017). The human body has an integrated antioxidant system comprising enzymatic and nonenzymatic antioxidants that help combat the harmful effects of ROS and RNS (Birben et al., 2012). Superoxide dismutase (SOD), catalase (CAT), and glutathione peroxidase (GPx) are the primary enzymatic antioxidants present in cells that help to protect cells from ROS-induced damage (Pizzino et al., 2017). The secondary enzymatic antioxidants, such as the thioredoxin system and glutaredoxins are important in maintaining cellular redox balance and repairing oxidized products (Birben et al., 2012). The nonenzymatic antioxidants would include low-molecular-weight compounds such as vitamins (vitamins A, C and E), β-carotene, uric acid (UA), α-lipoic acid, and glutathione (GSH), a tripeptide (L-g-glutamyl-L-cysteinyl-L-glycine) that comprise a thiol (sulfhydryl) group. While the primary antioxidants inhibit and scavenge oxidant formation, the other antioxidants in the body scavenge oxidants as well as repair the oxidized molecules (Vona et al., 2021).

An imbalance in the oxidant and antioxidant entities favouring the increase in oxidants, coupled with the body’s inability to salvage oxidized molecules, leads to oxidative stress. It has damaging effects on various cellular structures like proteins, lipids, and nucleic acids, which ultimately lead to various pathological conditions (Pizzino et al., 2017). Understanding the interplay between oxidant and antioxidant systems will help in studying oxidative stress-mediated diseases and will provide a rationale for improving therapeutic approaches to antioxidant defenses.

## Oxidant species

The fundamental process of energy production in the mitochondria is known to generate free radicals. When oxygen is used to produce adenosine triphosphate (ATP) in the body, ROS and RNS are produced as by-products owing to the cellular redox process (Pham-Huy et al., 2008). ROS and RNS are known as “free radicals.” These radicals possess one or more unpaired electrons in their outer shells. They are formed via the breakage of chemical bonds in a molecule such that each fragment keeps one electron, by cleavage of a radical to form another radical, and via redox reactions (Pham-Huy et al., 2008). Free radicals are highly unstable molecules that have unpaired electrons readily available to react with various organic substrates such as lipids, proteins, and DNA (Pham-Huy et al., 2008). Free radicals include O_2_^•^ˉ, ^•^OH, peroxyl (ROO^•^), nitric oxide (^•^NO), and nitrogen dioxide (^•^NO_2_) (Pham-Huy et al., 2008). On the other hand, the non-free radical species include H_2_O_2_, hypochlorous acid (HOCl), hypobromous acid (HOBr), ozone (O_3_), ^1^O_2_, nitrous acid (HNO_2_), nitrosyl cation (NO^+^), nitroxyl anion (NO^−^), dinitrogen trioxide (N_2_O_3_), dinitrogen tetraoxide (N_2_O_4_), nitronium (nitryl) cation (NO_2_^+^), hydroperoxides (ROOH), aldehydes (HCOR), and peroxynitrite (OONO^−^) (Phaniendra et al., 2015). These species can lead to free radical reactions in living organisms (Phaniendra et al., 2015).

### Production mechanisms of oxidant species

#### Free radical oxidants

Free radicals can be generated via enzymatic and non-enzymatic reactions. The superoxide anion radical (O_2_^•^ˉ) is a significant ROS generated enzymatically through the activity of xanthine oxidase, lipooxygenase, cyclooxygenase, and NADPH-dependent oxidase (Pham-Huy et al., 2008; Phaniendra et al., 2015). It can also be generated through nonenzymatic electron transfer reactions, where an electron is transferred to molecular oxygen (O_2_) (Phaniendra et al., 2015). This radical can exist in two states: O_2_^•^ˉ, under physiological pH or as hydroperoxyl radical (HO_2_) under low pH conditions (Phaniendra et al., 2015). The HO_2_ form is particularly crucial as it can easily traverse the phospholipid bilayer compared to the charged form (O_2_^•^ˉ). In a dismutation reaction (Equation 1), O_2_^•^ˉ can react with another O_2_^•^ˉ, wherein one radical is oxidized to O_2_ and the other is reduced to H_2_O_2_ (Phaniendra et al., 2015).

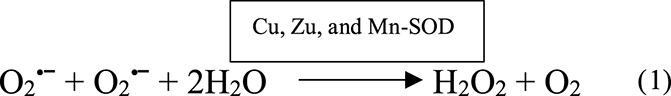




The hydroxyl radical (^•^OH) represents the neutral state of the hydroxide ion and serves as an extremely reactive free radical (Phaniendra et al., 2015). It arises through a Fenton reaction (Equation 2), where H_2_O_2_ interacts with metal ions like Fe^2+^ or Cu^+^ (Phaniendra et al., 2015). These metal ions are frequently bound within complexes with various proteins such as ferritin (an intracellular iron-storing protein), ceruloplasmin (a plasma copper-transporting protein), or other molecules (Phaniendra et al., 2015). During physiological stress, excessive O_2_^•^ˉ liberates free iron from ferritin. This liberated iron engages in the Fenton reaction to yield ^•^OH. Additionally, ^•^OH can be generated by the interaction between O_2_^•^ˉ and H_2_O_2_, termed the Haber-Weiss reaction (Equation 3) (Phaniendra et al., 2015). ^•^OH, exhibits potent reactivity towards both organic and inorganic compounds, including DNA, proteins, lipids, and carbohydrates (Birben et al., 2012; Phaniendra et al., 2015).

Fe2++H2O2 → Fe3++O•H+OH− Fenton reaction (2)

O2•ˉ+H2O2 →O•H+OH− Haber−Weiss reaction (3)

The peroxyl radical (ROO^•^) originates from O_2_ within biological systems. Its basic form is the per hydroxyl radical (HOO^•^), produced through the protonation of O_2_^•^ˉ. This radical has the potential to trigger lipid peroxidation (Equation 4) (Phaniendra et al., 2015).

$$O_{2}^{•}ˉ+H_{2}O\;⇌\text{HOO}•+{\text{OH}}^{-}$$ (4)

Nitric oxide synthases (NOS) convert L-arginine to L-citrulline in tissues to yield a small molecule called nitric oxide (^•^NO) (Equation 5). The reaction involves the oxidation of one of the terminal guanido nitrogen atoms to give ^•^NO (Phaniendra et al., 2015). There are three isoforms of NOS, including neuronal NOS (nNOS), endothelial NOS (eNOS), and inducible NOS (iNOS). All three forms aid in the formation of the ^•^NO. Because ^•^NO can dissolve in both water and lipids, it can easily diffuse through the cytoplasm and plasma membrane (Phaniendra et al., 2015). ^•^NO, is known to be a multifaceted molecule capable of having pro-oxidant as well as oxidant-protective effects. It is a crucial signalling molecule as it is a vasodilator that helps maintain endothelial function (Bloodsworth et al., 2000). It also has important immune functions, which will be discussed in detail in the later sections. The underlying oxidative status of a tissue is a key for determining ^•^NO function. If ^•^NO is in excess among other oxidants, then lipid oxidation and monocyte margination into the vascular wall will be attenuated, producing antiatherogenic effects. However, when endogenous tissue oxidant levels are high, ^•^NO can react with them to produce secondary oxidizing species that can promote membrane and lipoprotein lipid oxidation, which may further have proatherogenic effects (Bloodsworth et al., 2000).

L−Arginine+O2+NADPH → L−Citrulline+N•O+NADP+ (5)

Nitrogen dioxide (^•^NO_2_) is not generated as a free radical within the body. Instead, it is a prevalent environmental pollutant originating from external sources like combustion processes and bacterial activity (Zhouen et al., 1998). It is present in tobacco smoke. It can also form in aqueous environments through the acid breakdown of nitrite (NO_2_^−^) or exposure of nitrate (NO_3_^−^) or NO_2_^−^ solutions to ionizing radiation (Zhouen et al., 1998). Functioning as a potent oxidizing free radical, ^•^NO_2_ poses toxicity by inducing lipid peroxidation and is implicated in cell damage and subsequent cell death (Zhouen et al., 1998).

#### Non-free radical oxidant species

Hydrogen peroxide (H_2_O_2_) arises from the dismutation reaction facilitated by the enzyme SOD (Equation 1). Because it lacks a charge, it can readily diffuse through biological membranes, potentially leading to cellular harm. While it doesn't directly affect DNA, it can induce DNA damage by generating ^•^OH in the presence of transition metal ions (Phaniendra et al., 2015).

The powerful oxidant ozone (O_3_) is formed by the antibody-catalysed water oxidation pathway; an integral process occurring in all antibodies which is associated with inflammation (Phaniendra et al., 2015). O_3_ can form other reactive species and can lead to lipid peroxidation. It can oxidize different functional groups in proteins and nucleic acids, including amine, alcohol, HCOR, and sulphydryl (Phaniendra et al., 2015). O_3_ or O_3_-mediated free radicals can cause chromosomal aberrations (Phaniendra et al., 2015).

Singlet oxygen (^1^O_2_) is an electronically excited and meta-stable state of O_2_ (Phaniendra et al., 2015). The activation of neutrophils and eosinophils (Equation 6) or the enzymatic reactions catalysed by the enzymes, lipoxygenases, dioxygenases, and lactoperoxidase can lead to the formation of ^1^O_2_ (Phaniendra et al., 2015). It is formed when the O_2_ is excited to first state, ^1^Δ_g_, which is an extremely reactive state compared to the other higher electronically excited states (Phaniendra et al., 2015). It is a strong oxidizing agent, leading to DNA and tissue damage (Phaniendra et al., 2015).

HOCl+H2O2 →O12+H2O+Cl− (6)

Peroxynitrite (OONO^−^) is generated from the reaction between O_2_^•^ˉ and ^•^NO (Phaniendra et al., 2015; Radi, 2018) (Equation 7). Its reaction with carbon dioxide (CO_2_) forms the reactive nitroso peroxo carboxylate (ONOOCO_2_^−^) or peroxynitrous acid (ONOOH) (Phaniendra et al., 2015). Homolysis of ONOOH forms both ^•^OH and ^•^NO_2_. It may also rearrange to form NO_3_^−^. OONO^−^ oxidizes lipids, methionine, and tyrosine residues in proteins. Nitrotyrosine is a marker of OONO^−^ (Phaniendra et al., 2015). OONO^−^ also oxidizes DNA to form 8-nitroguanine, which is a marker of RNS-induced nitrative DNA damage (Phaniendra et al., 2015). These markers are discussed in the following sections.

O2•ˉ+N•O → OONO− (7)

The reaction of ^•^NO with O_2_ and H_2_O gives NO_3_^−^ and NO_2_^−^ ions. An electron oxidation of ^•^NO leads to the formation of a nitrosonium cation (NO_2_^∙^+), while an electron reduction results in ^•^NO. These ions can react with ^•^NO to yield N_2_O and OH^•^. ^•^NO reacts with radicals such as H_2_O_2_ and HOCl to give N_2_O_3_, ^•^NO_2_, and NO_3_^−^ (Phaniendra et al., 2015).

The halide oxidants hypochlorous acid (HOCl) and hypobromous acid (HOBr) are produced from H_2_O_2_, and the corresponding halide ions (Cl^−^ and Br^−^) catalysed by the leukocyte-derived heme peroxidase enzymes myeloperoxidase (MPO) and eosinophil peroxidase (EPO), respectively (Rees et al., 2010; Wu et al., 1999). HOCl has important antibacterial properties and aids in immune function (Boecker et al., 2023). It engages in a critical immune process known as “respiratory burst.” However, due to its high reactivity, it can oxidize various biological molecules such as thiols, ascorbate, urate, pyridine nucleotides, and tryptophan. It chlorinates numerous substances, including amines to form chloramines, tyrosyl residues to yield ring chlorinated products, cholesterol, and unsaturated lipids to produce chlorohydrin. Additionally, it has the capability to chlorinate DNA (Phaniendra et al., 2015). Similarly, HOBr readily reacts with amino acids, proteins, antioxidants like thiols, carbohydrates, lipids, and DNA (Pattison and Davies, 2004).

#### Sources of oxidant species

Oxidant species can originate from either “endogenous” or “exogenous” origins. Endogenous sources include various cellular organelles such as mitochondria, peroxisomes, and endoplasmic reticulum, where oxygen consumption rates are elevated. The cytosol and plasma membrane also contribute to endogenous production of oxidant species (Di Meo et al., 2016). Exogenous sources include external entities such as toxins, UV radiation, alcohol, tobacco smoke, certain medications, and so on (Vona et al., 2021).

### Endogenous sources

#### Production of ROS

##### Metabolism

###### Mitochondria

The mitochondria are the organelles that produce the highest amount of intracellular ROS. They contribute to approximately 90% of cellular ROS generated in the body (Tirichen et al., 2021). 0.2%–2.0% of the O_2_ consumed by mitochondria is reduced to O_2_^•^ˉ (Tirichen et al., 2021). Complex I (NADH dehydrogenase) and complex III (ubiquinone cytochrome c reductase) are the two major sites in the electron transport chain that produce super O_2_^•^ˉ. When electrons are transferred from complex I or II to coenzyme Q or ubiquinone (Q), the reduced form of coenzyme Q (QH_2_) is formed. This reduced form of QH_2_ regenerates coenzyme Q via an unstable intermediate semiquinone anion (^∙^Q^−^) in the Q-cycle. An immediate transfer of electrons from the formed ^∙^Q^−^ to O_2_ yields O_2_^•^ˉ. As this generation of O_2_^•^ˉ is non-enzymatic, it has a higher metabolic rate, which leads to a greater production of ROS (Frei, 1994).

Other components within the mitochondria that contribute to the generation of ROS are monoamine oxidase, α-ketoglutarate dehydrogenase, glycerol phosphate dehydrogenase, and p66shc (Phaniendra et al., 2015). Belonging to the adaptor protein family, p66Shc plays roles in regulating lifespan and apoptosis (Xu et al., 2020). While predominantly found in the cytoplasm, a fraction of p66Shc resides in the mitochondrial intermembrane space, where it can initiate ROS production. During periods of oxidative stress, p66Shc relocates to the mitochondrial intermembrane space, where it interacts with cytochrome-c, consequently fostering ROS generation (Phaniendra et al., 2015).

###### Peroxisomes

The respiratory pathway in peroxisomes involves the transfer of electrons from various metabolites to O_2_, which leads to the formation of H_2_O_2_. The β-oxidation of fatty acids is the major process producing H_2_O_2_ in the peroxisomes (Phaniendra et al., 2015). The β-oxidation enzymes, acyl CoA oxidases, D-amino acid oxidase, L-α-hydroxy oxidase, urate oxidase, and D-aspartate oxidase produce H_2_O_2_ while xanthine oxidase produce H_2_O_2_, O_2_^•^ˉ, and ^•^NO (Phaniendra et al., 2015; Fransen et al., 2012). The H_2_O_2_ inside peroxisomes may give rise to ^•^OH through the Fenton reaction. The presence of ^•^NO and O_2_^•^ˉ kinetically and thermodynamically favours their reaction to form OONO^−^ in the peroxisomes (Fransen et al., 2012).

###### Endoplasmic reticulum

Within the endoplasmic reticulum, metabolic enzymes such as cytochrome p-450 and b5, along with diamine oxidase, play roles in generating ROS. Additionally, the thiol oxidase enzyme, Erop1p, facilitates the transfer of electrons from dithiols to O_2_, leading to the formation of H_2_O_2_ (Phaniendra et al., 2015).

###### Cytosol

In the cytosol, ROS can be formed via NADPH activity and can influence metabolic processes including glycolysis and downstream oxidative phosphorylation, pentose phosphate pathway activity, and autophagy (Forrester et al., 2018).

###### Plasma membrane

The plasma membrane made up of the lipid bilayer is also crucial in producing free radicals as it is generally exposed to an oxidizing environment (Di Meo et al., 2016). The production of O_2_^•^ˉ by phagocytic cells occurs via the plasma membrane-localized, NADPH oxidase (NOX) (Fisher, 2009). Free radicals formed from the plasma membrane can, in turn, attack the fatty acyl chain or the head group of phospholipids in the lipid bilayer. ROS can also target the side chains of membrane proteins. ROS abstracting hydrogen from membrane lipids further leads to the formation of ROS, which, upon reaction with O_2_, gives rise to peroxide-containing products. Hydrogen abstraction of unsaturated acyl chains can initiate a chain reaction that propagates to other lipids present in a bilayer. This reaction is generally amplified and can result in the formation of many lipid peroxides (Wang et al., 2017).

#### Inflammation

Inflammation is the body’s primary immune response to invading pathogens or foreign substances. In the innate immune system, macrophages are essential for eliminating pathogens by generating reactive species such as O_2_^•^ˉ, H_2_O_2_, ^•^OH, ^•^NO, OONO^−^, and HOCl. This process continues until the pathogens are eliminated and repair mechanisms are completed. However, prolonged inflammation can cause cell damage or hyperplasia due to excessive ROS production from inflammatory cells. Chronic inflammation allows ROS to interact with DNA in dividing cells, leading to recurrent DNA damage and a higher likelihood of genomic mutations (Khansari et al., 2009). Additionally, these ROS can damage lipids and proteins in the body.

Other sources of endogenous free radicals can be mental stress, excessive exercise, ischemia, cancer, and aging (Pham-Huy et al., 2008).

### Production of RNS

The enzymes NOS catalyse the conversion of L-arginine into L-citrulline and ^•^NO by 5-electron oxidation of the guanidine nitrogen of L-arginine (Di Meo et al., 2016). NOS exists in multiple isoforms and is found in various cell types, predominantly located in either the plasma membrane or cytosol of these cells. To date, there are 3 known isoforms of NOS: nNOS; type I NOS, eNOS; type III NOS, and iNOS; type II NOS (Di Meo et al., 2016). nNOS synthesizes ^•^NO in neurons where it aids in communication between nerve cells, whereas ^•^NO generated by iNOS in macrophages and smooth muscle cells contributes to their killing action (Di Meo et al., 2016). The endothelium, brain, and heart also produce ^•^NO via eNOS, where ^•^NO regulates blood pressure (Di Meo et al., 2016).

### Exogenous sources of ROS and RNS

#### Cigarette smoke and alcohol

Cigarette smoke contains many free radicals, including O_2_^•^ˉ and ^•^NO. Additionally, the inhalation of cigarette smoke into the lungs also activates various endogenous mechanisms, such as the accumulation of neutrophils and macrophages, which further contribute to oxidant injury (Birben et al., 2012). Alcohol (chemically known as ethyl alcohol or ethanol) is commonly consumed across the globe. A deleterious effect of ethanol metabolism is its implications in oxidative stress. Ethanol is broken down in the liver in two steps: first, it is metabolized to acetaldehyde. Next, the enzyme aldehyde dehydrogenase converts acetaldehyde to acetate. Both reactions produce a molecule of NADH. This provides more starting material for the respiratory chain reaction and, therefore, increased production of O_2_^•^ˉ (Wu and Cederbaum, 2003). Systems producing O_2_^•^ˉ will subsequently result in the formation of H_2_O_2_ (Wu and Cederbaum, 2003).

#### Ozone (O_3_)

O_3_ exposure can lead to lipid peroxidation. It can also induce an influx of neutrophils into the airway epithelium, which accelerates oxidant injury (Birben et al., 2012). Even short-term exposure to O_3_ can result in the release of inflammatory mediators such as MPO, eosinophil cationic proteins, lactate dehydrogenase, and albumin. These factors can contribute to oxidative stress (Birben et al., 2012).

#### Ionizing radiation

In the presence of O_2_, ionizing radiation converts ^•^OH, O_2_^•^ˉ, and organic radicals to H_2_O_2_ and ROO^•^. These ROO^•^ species then react with the active redox metal ions, Fe^2+^ and Cu^+^, via Fenton reactions, leading to oxidative stress (Birben et al., 2012). Oxidative reactions are triggered by ultraviolet A (UVA) photons owing to the excitation of endogenous photosensitizers, such as porphyrins, NOX, and riboflavin. 8-Oxo-7,8- dihydroguanine (8-oxoGua) is the main UVA-mediated DNA lesion product formed by the oxidation of ^•^OH, 1-electron oxidants, and ^1^O_2_ that mainly reacts with guanine (Birben et al., 2012). Ionizing radiation can effectively bring about the formation of the guanine radical cation (Birben et al., 2012).

#### Xenobiotics

Oxidative stress is believed to be the most common mechanistic feature in toxicology (Samet and Wages, 2018). The physio-chemical properties of various xenobiotics, including heavy metals, environmental toxins, and per- and polyfluoroalkyl substances (PFAS), are known to induce oxidative stress (Samet and Wages, 2018). Heavy metals, including iron, copper, cadmium, mercury, nickel, lead, and arsenic, can generate free radicals, resulting in cellular damage. Generally, metal-mediated free radical production is brought about by the Fenton or Haber-Weiss reactions (Equations 8, 9). Due to these reactions, metals like iron and copper can react with H_2_O_2_ and O_2_^•^ˉ to give ^•^OH (Birben et al., 2012).

$${\text{Metal}}^{3+}+O_{2}\;\to \;{\text{Metal}}^{2+}+O_{2}\;\left(\text{Haber}-\text{Weiss}\right)$$ (8)

Metal2++H2O2 → Metal3++OH−+O•H Fenton reaction (9)

Apart from these reactions, certain metal ions directly react with cellular molecules to generate free radicals, such as thiol radicals (Birben et al., 2012). These radicals may also react with other thiol molecules to generate O_2_^•^ˉ. O_2_^•^ˉ can further be converted to H_2_O_2_. Some metals, such as arsenite, induce ROS production indirectly by activating the radical-producing systems in cells (Birben et al., 2012). Arsenic is a highly toxic element as it not only generates a variety of oxidants (^•^OH, ^1^O_2_, ROO^•^, ^•^NO, H_2_O_2_, and dimethylarsinic peroxyl radicals) but also inhibits numerous antioxidant enzymes (including the GSH-dependent enzymes, such as glutathione-S-transferases (GST), GPx, and glutathione reductase (GR), via binding to their sulfhydryl (-SH) group) (Birben et al., 2012). The metal lead can cause lipid peroxidation. It is known to significantly decrease the activity of tissue SOD and brain GPx (Birben et al., 2012).

Environmental toxins such as bisphenol A (BPA) are known to give rise to oxidative stress-mediated metabolic and hormonal disturbances (Meli et al., 2020). The chemical, once inhaled or ingested from the environment or common consumer products, mainly gets metabolized into bisphenol A glucuronide (BPAG) or bisphenol A sulfate (BPAS) and is eliminated through urination (Fisher, 2009). However, a portion of the remaining free BPA in the body can produce ROS via the enzymatic (H_2_O_2_/peroxidase and NADPH/CYP450) and non-enzymatic (OONO^−^/CO_2_ and −OCl/HOCl) formation of phenoxyl radicals. Subsequently, these radicals react with NADPH or intracellular GSH to produce a variety of radical species, including O_2_^•^ˉ, peroxides, and ^•^OH, thereby leading to oxidative stress (Meli et al., 2020).

PFAS are commonly found in a wide range of consumer goods. These goods release PFAS, and they persistently remain in the environment (Taibl et al., 2022). PFAS can be ingested from contaminated food and water. This can increase the burden of PFAS in the body, leading to oxidative stress (Taibl et al., 2022). Exposure to PFAS is believed to overwhelm and destabilize the mitochondria, which limits its effectiveness in managing ROS, thereby resulting in oxidative stress (Taibl et al., 2022).

#### Medications

**FIGURE 1:**
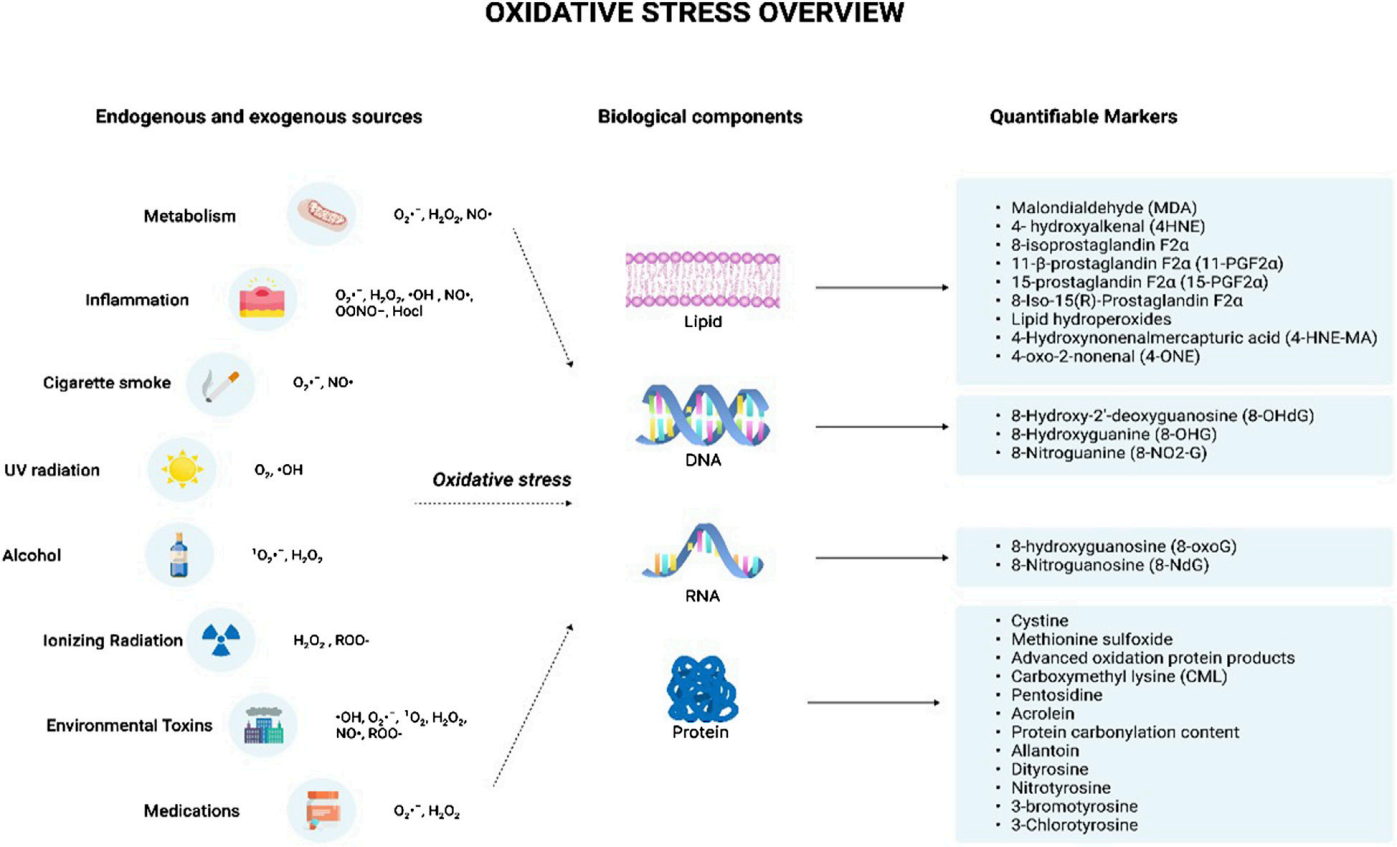
Oxidative stress overview. The illustration indicates the various sources that can trigger the production of reactive oxygen species and reactive nitrogen species. The endogenous sources include metabolism and inflammation while the exogenous sources include cigarette smoke, UV radiation, alcohol, ionizing radiation, environmental toxins, and medications. These sources lead to the production of various free radicals such as O_2_^·^ˉ, H_2_O_2_, ^·^OH, NO^·^, OONO−, Hocl, ^1^O_2_, and ROO- which can give rise to oxidative stress. This results in the damage of various cellular components including lipids, DNA, RNA, and protein leading to the formation of damaged products. The damaged products act as good markers of oxidative stress indicating the degree of oxidative stress-mediated damage for each component. Abbreviations: O_2_^·^ˉ, Superoxide anion radical; H_2_O_2_, Hydrogen peroxide; ^·^OH, Hydroxyl radical; NO^·^, Nitric oxide; OONO−, Peroxynitrite; Hocl, Hypochlorous acid; ^1^ O_2_, Singlet molecular oxygen; ROO-, Hydroperoxides; DNA, Deoxyribonucleic acid; RNA, Ribonucleic acid.

Certain immunosuppressant drugs, such as cyclosporine, tacrolimus, and gentamycin, are known to contribute to oxidative stress as they increase free radical levels via lipid peroxidation (Pizzino et al., 2017). The drug, Doxorubicin (Dox), is an anthracycline antibiotic used as a chemotherapeutic agent. The drug can react with mitochondrial reductases to readily reduce O_2_ to O_2_^•^ˉ, and H_2_O_2_. The reactions between Dox and iron can also produce ROS, and this reaction can subsequently generate an iron II-Dox free radical capable of reducing O_2_ (Deavall et al., 2012). The antineoplastic agent, cisplatin used in the treatment of testicular, bladder, lung, gastrointestinal, and ovarian cancers is also seen to increase oxidative stress by increasing levels of O_2_^•^ˉ, H_2_O_2_, and ^•^OH (Deavall et al., 2012). A class of drugs called ‘pro-oxidants’ use their ability to induce oxidative stress to kill cancer cells. It is known that cancer cells are more sensitive to oxidative stress than normal cells. Therefore, pro-oxidant cancer drugs dramatically increase intracellular ROS and thus, induce oxidative stress by interfering with ROS homeostatic regulators such as glutathione S-transferase pi 1 (GSTP1) (Choi et al., 2019). Figure 1 indicates the major endogenous and exogenous sources that can give rise to oxidative stress, resulting in damage to biological components.

### Measuring oxidative stress

#### Direct quantification of oxidant species

ROS and RNS are the key players responsible for the deleterious effects of oxidative stress. Direct quantification of their levels is one approach of determining oxidative stress (Katerji et al., 2019).

#### H_2_O_2_, ^•^OH and ROO^•^

**FIGURE 2:**
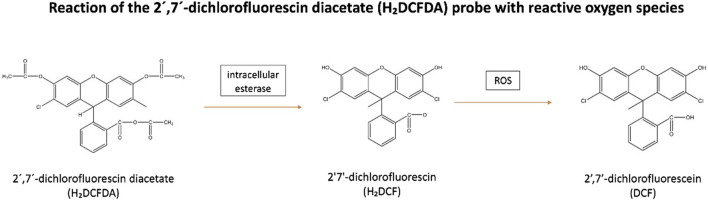
Reaction of the 2′,7′-dichlorofluorescin diacetate (H₂DCFDA) probe with reactive oxygen species. H₂DCFDA enters cells and is hydrolyzed by intracellular esterase to form non-fluorescent, H₂DCF. H₂DCF remains in the cells and reacts with H₂O₂ to produce fluorescent, DCF. Abbreviations: H₂DCFDA, 2′7′-dichlorofluorescin diacetate; H₂DCF, 2′7′-dichlorofluorescin; DCF, 2′,7′-dichlorofluorescein; ROS, Reactive oxygen species.

These reactive species can be measured following staining with 2′7′-dichlorofluorescin diacetate (H₂DCFDA). This membrane-permeable fluorogenic probe diffuses into the cells where it becomes hydrolysed by intracellular esterase to form the non-fluorescent, 2′7′-dichlorofluorescin (H₂DCF). H₂DCF remains trapped within the cells and reacts with H_2_O_2_, generating the fluorescent, 2′,7′-dichlorofluorescein (DCF) (Nova et al., 2020) (Figure 2). The amount of cellular H_2_O_2_ can be estimated by the fluorescence intensity of DCF (λ _excitation_ = 488 nm and λ _emission_ = 530 nm) which be analyzed by flow cytometry or via a fluorescence plate reader (Katerji et al., 2019). However, it has been observed H₂DCF is not only oxidative by H_2_O_2_ to give DCF, but also by other ROS. This makes the probe non-specific to H_2_O_2_ (Murphy et al., 2022). Additionally, this reaction is sensitive to local O_2_ levels and pH, implying that the fluorescence yield may not be linear with increased ROS levels (Murphy et al., 2022).

**FIGURE 3:**
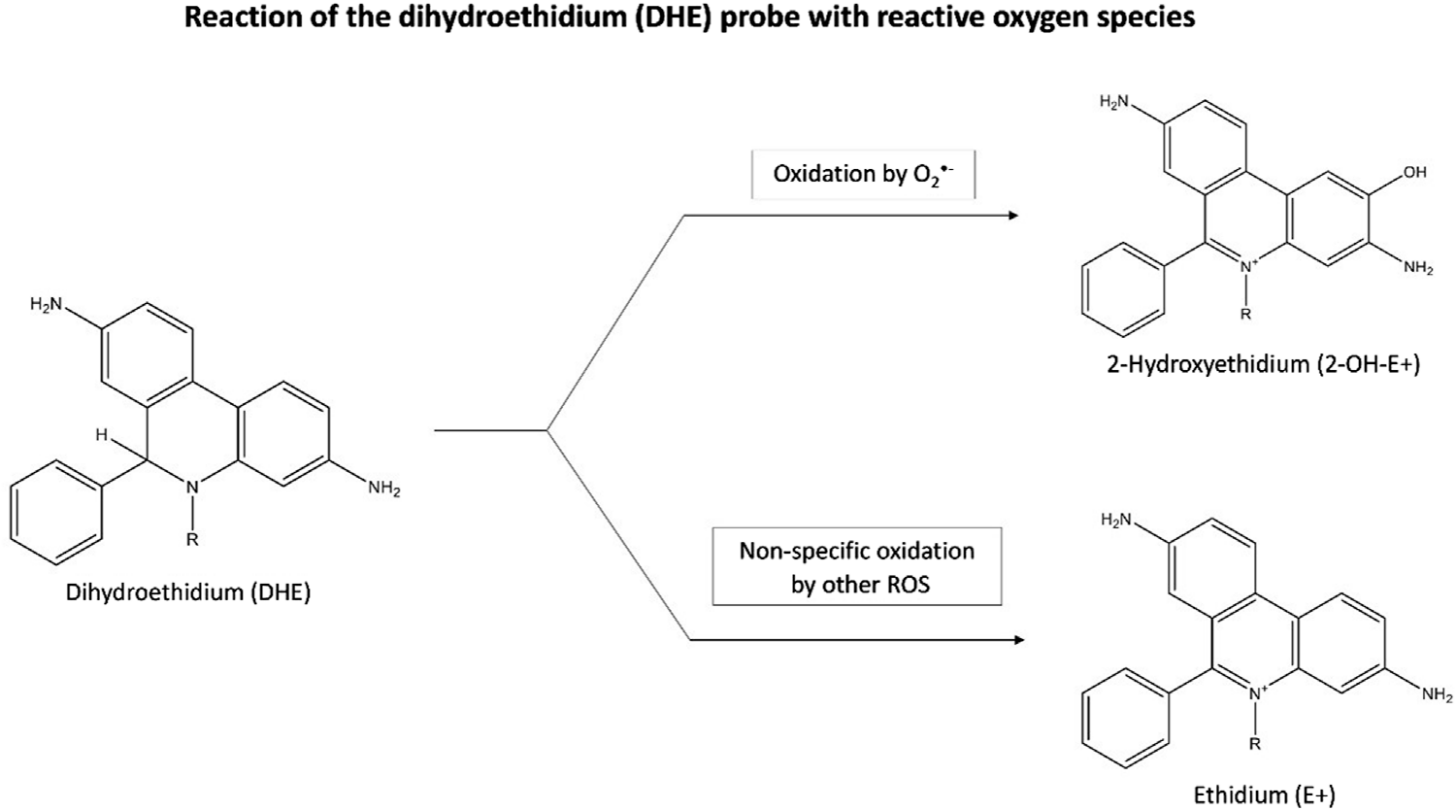
Reaction of the dihydroethidium (DHE) probe with reactive oxygen species. Inside cells, DHE is oxidized by O_2_^·^ˉ to form fluorescent 2-OH-E+, with red fluorescence indicating O_2_^·^ˉ levels. DHE can also be non-specifically oxidized by other oxidants like H_2_O_2_, and ^·^OH, producing fluorescent E+. Abbreviations: DHE, Dihydroethidium; 2-OH-E+, 2-hydroxyethidium; E+, Ethidium; O_2_^·^ˉ, Superoxide anion radical; ROS, Reactive oxygen species.

O_2_^•^ˉ can be quantified from staining with the fluorescent probe, dihydroethidium (DHE). Inside the cells, DHE is directly oxidized to 2-hydroxyethidium (2-OH-E+) by O_2_^•^ˉ, which then fluoresces (Villaverde et al., 2019) (Figure 3). A flow cytometer or a fluorescence plate reader can then measure the red fluorescence (λ _excitation_ = 488 nm and λ _emission_ = 585 nm) which is proportional to the intracellular O_2_^•^ˉ levels (Katerji et al., 2019) However, this quantification can be misleading as DHE is also susceptible to non-specific oxidation by other oxidants such as H_2_O_2_, and ^•^OH, generating ethidium (E+) (Figure 3). As the 2 products, 2-OH-E+ and E+ have overlapping fluorescence spectra, it is difficult to differentiate the contribution of non-specific oxidation and O_2_^•^ˉ-dependent oxidation (if any) to the overall fluorescence (Murphy et al., 2022).

Direct quantification of ROS levels with high accuracy and precision in biological species is tedious owing to their short lifespan. While H_2_O_2_ (chemically stable) and ROO^•^ (7s) are relatively stable molecules with half-lives of seconds to minutes, the other oxidant species such as ^•^OH (10^–9^ s), O_2_^•^ˉ (10^–6^ s), alkoxyl anions (10^–6^ s), and ^1^O_2_ (10^–6^ s) are very reactive having half-lives of less than a nanosecond (Katerji et al., 2019; Rubio and Cerón, 2021). This makes it difficult to measure them in biological samples. Although the levels of oxidant species are high during oxidative stress, their levels are still lower than those of other cellular components, which makes their quantification difficult using conventional methods (Murphy et al., 2022). ROS are highly reactive and are continuously reacting with cellular components to yield new molecules, such as lipid peroxidation products or protein carbonyls, which are now studied as indirect markers of oxidative stress. Also, the body is bestowed with antioxidants, which constantly aim at quenching free radicals. Therefore, it becomes challenging to measure ROS directly without considering the impact of antioxidant systems. Attempts have been made to quantify ROS using complex techniques such as electron spin resonance, spin trapping, or pulse radiolysis (Kehm et al., 2021). However, these techniques can be labour-intensive, time-consuming, and may require sophisticated instrumentation, which limits their general use (Rubio and Cerón, 2021). The simpler spectrophotometric techniques are unable to measure various ROS; they are non-specific to individual ROS and can only measure the relatively stable ROS (Rubio and Cerón, 2021).

As the direct quantification of ROS is fraught with various limitations and challenges, indirect means of detecting oxidative stress have been utilized. The indirect markers include markers of lipid peroxidation, nucleic acid, and protein damage, which will indicate the level of oxidative stress based on the damage done to these cellular components. Additionally, the quantification of antioxidants in the body is also quantified to assess the body’s ability to counteract oxidative stress, with insufficient antioxidant levels being indicative of oxidative stress. The markers under either category will be discussed in the later sections.

### Beneficial functions of oxidant species

Oxidant species are seen to play dual roles by benefiting the body at lower levels and being harmful at higher levels (Pham-Huy et al., 2008). The finding that the ^•^OH radical helps stimulate the production of cyclic guanosine monophosphate (cGMP) (a signalling messenger molecule) has led to an understanding of the dual nature of ROS and RNS in biological systems (Phaniendra et al., 2015). It then became clear that the human body not only adapted to a coexistence with free radicals but also developed means to utilize these toxicants to their own advantage by using them in critical physiological processes (Phaniendra et al., 2015). This has been supported by the fact that at low or moderate concentrations, ROS regulate cell growth and apoptosis at the cellular level (Phaniendra et al., 2015). ROS can contribute toward cell survival in two ways: by either acting on transcription factors that directly interact with specific DNA motifs on promoters of target genes or via the activation of mitogen-activated protein kinases (MAPK), phosphoinositide 3-kinases (PI3Ks), phosphatase and TENsin homolog (PTEN), and protein tyrosine phosphatases that initiate signalling in several cellular processes, including proliferation and survival (Di Meo et al., 2016).

At the system level, ROS contributes to complex functions, such as immune function. Phagocytes such as neutrophils, macrophages, and monocytes release free radicals to destroy invading pathogens (Pham-Huy et al., 2008). During bacterial infection, these cells identify and engulf bacteria, leading to the formation of a vesicle called the phagosome. This process activates the otherwise dormant enzyme present in the cytosol and plasma membrane, NOX. This activation is brought about by cytochrome b558 and the translocation of the cytosolic components to the phagosome membrane (Di Meo et al., 2016). Phagosome maturation is mediated by the successive fusion and fission interactions between the new phagosome and early endosomes, late endosomes, and finally lysosomes, leading to the formation of the ‘phagolysosome.’ The phagolysosome is the final microbicidal organelle, and it contains hydrolytic enzymes (cathepsins, proteases, lysozymes, and lipases) and scavenger molecules, including NOX (Rosales and Uribe-Querol, 2017).

At this stage, the catalytically activated NOX undergoes a ‘respiratory burst’ wherein it uses up enormous amounts of O_2_ to produce O_2_^•^ˉ. This O_2_^•^ˉ then dismutates to H_2_O_2_, which can in turn react with O_2_^•^ˉ to generate more-complex ROS such as ^•^OH and ^1^O_2_ (Rosales and Uribe-Querol, 2017). Additionally, H_2_O_2_ can be combined with Cl^−^ ions to give HOCl via the enzyme, MPO (Rosales and Uribe-Querol, 2017). These ROS being highly reactive, damage the bacterial proteins, lipids, and nucleic acids, thereby disrupting the bacterium’s vital functions. HOCl particularly has antimicrobial functions and can further damage bacterial components, leading to bacterial death (Cross and Segal, 2004). The critical role of ROS in immune function has been supported by their absence in granulomatous disease patients. These patients have an impaired membrane-bound NOX system which makes them unable to produce the O_2_^•^ˉ, resulting in persistent infections (Drummond et al., 2011).

The respiratory burst is the only physiological mechanism that produces HOCl, which can then react with tyrosyl residues in proteins to give 3-chlorotyrosine (Buss et al., 2003). Therefore, 3-chlorotyrosine has emerged as a specific marker for the oxidant activity of MPO-containing cells (Buss et al., 2003). As 3-chlorotyrosine results from phagocytic activity only, a rise in its levels could also be indicative of increased phagocytosis owing to persistent infection. This may justify the elevated levels of 3-chlorotyrosine observed in infants who had lung infections or were Ureaplasma urealyticum positive (Buss et al., 2003). From this, we suggest that 3-chlorotyrosine not only serves as a biomarker of the oxidant activity of MPO-containing cells but also as a marker of infection.

Interestingly, ROS are also involved in the expression of antioxidants. This is mediated by the expression of the transcription factor nuclear factor erythroid 2-related factor 2 (Nrf2), which regulates the expression of several antioxidant and detoxifying genes by binding to promoter sequences containing a consensus antioxidant response element (Di Meo et al., 2016). ROS initiate the Nrf2-Keap1 (Kelch-like ECH-associated protein 1) pathway by modifying critical cysteine residues of Keap1 and Nrf2. This results in the activation of the Nrf2-controlled genes that encode detoxification enzymes NQO1 (NAD(P)H quinone oxidoreductase 1), antioxidant enzymes (GPx2, Srx1 (Sulfiredoxin 1)), and enzymes that synthesize low-molecular-weight antioxidants (GSH, bilirubin), all of which suppress oxidative stress (Ma, 2013).

In a similar manner, the RNS, ^•^NO has significant functions within the body. It acts as an intracellular second messenger, activating guanylate cyclase and protein kinases. It also helps relax smooth muscles in blood vessels and serves as a cellular redox regulator by modifying enzymatic activity through protein nitrosylation. (Phaniendra et al., 2015). ^•^NO is also crucial for nonspecific host defense and for destroying intracellular pathogens and tumors (Pham-Huy et al., 2008). It does so by regulating the growth, function, and death of crucial immune cells, including macrophages, T lymphocytes, antigen-presenting cells, mast cells, neutrophils, and natural killer cells (Coleman, 2001). ^•^NO is also believed to have a potential microbicidal effect via the reaction of ^•^NO with iron or thiol groups on proteins forming iron-nitrosyl complexes. These complexes can induce nitrative stress in the microbial cells, which can lead to cell death (Coleman, 2001). In conclusion, ROS and RNS are continuously produced owing to metabolic activities, and they are vital to human health at low or moderate levels.

### Detrimental effects of oxidant species

An imbalance between the formation and neutralization of ROS and RNS species, favoring their high levels, leads to ‘oxidative stress.’ Under such conditions, the oxidant species attack biological components such as lipids, nucleic acids, and proteins (Pham-Huy et al., 2008). The mechanism of the damaging effects of oxidant species on these cellular structures has been discussed below.

#### Lipids

Polyunsaturated fatty acid (PUFA) residues of phospholipids are most susceptible to oxidation by free radicals (Phaniendra et al., 2015). These membrane lipids can undergo lipid peroxidation, leading to impaired membrane function, such as reduced fluidity, and the deactivation of enzymes and receptors embedded in the membrane. (Phaniendra et al., 2015). Lipid peroxidation is a chain mechanism and involves three events: initiation, propagation, and termination. An initiating free radical, which can be hydroxyl, alkoxyl, ROO^•^, or OONO^−^, can oxidize numerous lipid molecules through sequential, self-propagating chain reactions (Milne et al., 2011). Of the mentioned free radicals, the ^•^OH is the most active and is likely to initiate the peroxidation process. The catalytic metal ions, copper (Cu^I^) or iron (Fe^II^) also aid in initiating the chain reaction (Milne et al., 2011). Lipid peroxidation is initiated when a free radical attacks hydrogen from a methylene group (CH_2_) in a fatty acid which results in the formation of a carbon-centered lipid radical (L^•^). This L^•^ then reacts with O_2_ to form a lipid peroxyl radical (LOO^•^), which undergoes rearrangement through a cyclization reaction to form endoperoxides. PUFAs such as linoleic acid (LA) (18:2), arachidonic acid (AA) (20:4), eicosapentaenoic acid (EPA) (20:5), and docosahexaenoic acid (DHA), are targets of free radical-initiated lipid peroxidation, yielding a diverse array of products (Milne et al., 2011). The rate at which these PUFAs get oxidized is subject to the number of -CH_2_- centres in the molecule that are flanked by two double bonds (bisallylic methylene) (Milne et al., 2011).

The primary products of free radical-initiated peroxidation of PUFAs are lipid hydroperoxides (LOOH). Oxidation of linoleates yields hydro (pero)xyoctadienoates (H(P)ODEs) (Milne et al., 2011). The decomposition of LOOHs yields the HCORs, acrolein, malondialdehyde (MDA), and 4-hydroxy-2-nonenal (4-HNE). MDA and 4-HNE are toxic lipid peroxidation products as they can damage the DNA and proteins (Phaniendra et al., 2015). These products can further propagate the peroxidation process by extracting hydrogen atoms from the other lipid molecules. MDA and 4-HNE have risen as important biomarkers of lipid peroxidation. The other lipid peroxidation products, 4-Hydroxynonenalmercapturic acid (4-HNE-MA) and 4-oxo-2-nonenal (4-ONE) can also be used as biomarkers. MDA and 4-HNE can undergo nucleophilic reaction of proteins with reactive carbonyl species to yield advanced lipoxidation end products. One such important reaction is their reaction with lysine residue proteins to form carboxymethyl lysine (CML), which has risen as an important marker of oxidative stress (Fu et al., 1996).

Secondary lipid peroxidation products are generated from the non-enzymatic free radical-catalysed peroxidation of AA and other highly unsaturated PUFAs. These secondary lipid peroxidation products include a series of prostaglandin (PG)-like products termed isoprostanes (IsoPs) (Phaniendra et al., 2015; Milne et al., 2011). They are important targets of lipid peroxidation of AA. The abstraction of a bisallylic hydrogen atom and the addition of a molecule of O_2_ to AA yields a ROO^•^. Following this, the ROO^•^ undergoes 5-exo cyclization and a second molecule of O_2_ gets added to the backbone of the compound to form PGG2-like compounds. F2-isoprostanes (F2-IsoP) is a subclass of IsoPs. The unstable bicycloendoperoxide PGG2-like intermediates are then reduced to give the four F2-IsoP regioisomers, namely, the 5, 8, 12, and 15 regioisomer series of F2-IsoP, depending on the carbon atom to which the allylic hydroxyl is attached (Murphy et al., 2022). The four F2-IsoP regioisomers, each comprises eight racemic diastereomers and depending of the combination of the isomers, they can generate 64 possible compounds (Galano et al., 2013). 8-, 9-, 11-, and 12-peroxy radicals of AA are known to make up the F2-IsoPs class (Ito et al., 2019). They are the intermediates generated during the formation of the above-mentioned F2-IsoP regioisomers. The F2-IsoPs class of AA are “gold standard” biomarkers of endogenous lipid peroxidation and oxidative stress (Phaniendra et al., 2015; Milne et al., 2011). Although there are assays for various IsoPs, 8-isoprostaglandin F_2_α (8-isoPGF_2_α, also known as 8-epi-PGF_2_α or 8-isoprostane; 15-F2t-IsoP) is commonly assessed as a biomarker of oxidative stress. Additionally, there are other F2-IsoPs products such as 11-β-prostaglandin F2α (11-PGF2α) and 15-prostaglandin F2α (15-PGF2α) as well as the isomer of 8-isoPGF2α, 8-Iso-15(R)-Prostaglandin that are quantified as biomarkers of lipid peroxidation. Table 1 summarizes the quantification techniques for the established lipid peroxidation markers.

**TABLE 1:** Markers of lipid peroxidation.

Lipid peroxidation markers			
Marker	Sample	Technique	Reference
Lipid hydroperoxides (LOOH)	Tissue, plasma, serum lipoproteins	Chemiluminescence-Based HPLC Detection Iodometric Assay Ferrous Oxidation of Xylenol	Moore and Roberts (1998), Sochor et al. (2012)
Malondialdehyde (MDA)	Serum, plasma, urine, CSF, erythrocytes, saliva	TBARS assay* HPLC GC-MS	Kehm et al. (2021)
4-hydroxy-2-nonenal (4-HNE)	Serum, plasma, urine, CSF, tissue	GC-MS* HPLC–MS/MS ELISA IHC	Kehm et al. (2021), Stopforth et al. (2006), Martinez-Moral and Kannan (2022)
4-Hydroxynonenalmercapturic acid (4-HNE-MA)	Urine	HPLC–MS/MS	Martinez-Moral and Kannan (2022)
4-oxo-2-nonenal (4-ONE)	Urine	Isotope-dilution mass spectrometry	Kuiper et al. (2010)
8-isoprostaglandin F2α	Urine	GC-MS* ELISA HPLC–MS/MS	Ito et al. (2019), Martinez-Moral and Kannan (2022)
11-β-prostaglandin F2α (11-PGF2α)	Urine	HPLC–MS/MS	Martinez-Moral and Kannan (2022)
15-prostaglandin F2α (15-PGF2α)	Urine	HPLC–MS/MS	Martinez-Moral and Kannan (2022)
8-Iso-15(R)-Prostaglandin F2α	Urine	HPLC–MS/MS	Martinez-Moral and Kannan (2022)

*Indicates the “gold standard technique” for a given marker.

Abbreviations: HPLC, High-Performance Liquid Chromatography; TBARS, Thiobarbituric acid reactive substances; GC-MS, Gas Chromatography-Mass Spectrometry; HPLC–MS/MS, High-Performance Liquid Chromatography with Tandem Mass Spectrometry; ELISA, Enzyme-Linked Immunosorbent Assay; IHC, Immunohistochemistry.

#### Nucleic acids

ROS and RNS can oxidatively damage nucleic acids resulting in base substitution, addition, deletion, and other mutations (Guo et al., 2017). The oxidative damage caused to DNA and RNA are discussed below.

#### Deoxyribonucleic acid (DNA)

ROS, particularly the ^•^OH radical reacts directly with the various components of DNA including the purine and pyrimidine bases, and the deoxyribose sugar backbone. This results in a number of alternations including single and double-stranded breaks in DNA (Phaniendra et al., 2015). When the ^•^OH radical attacks pyrimidine by removing hydrogen atoms, it generates various pyrimidine derivatives, such as thymine glycol, uracil glycol, 5-hydroxydeoxyuridine, 5-hydroxydeoxycytidine, and hydantoin, among others (Phaniendra et al., 2015). Similarly, the attack of ^•^OH radical on purine results in the formation of 8-Hydroxy-2′-deoxyguanosine (8-OHdG), 8-hydroxy deoxy adenosine, and 2,6-diamino-4-hydroxy-5-formamidopyrimidine (Phaniendra et al., 2015). More specifically, when guanine gets oxidized by ^•^OH radical, a ^•^OH is added to the eighth position of the purine base leading to the formation of the oxidatively modified product, 8-OHdG (Verigos et al., 2020).

8-OHdG is an important biomarker of oxidative DNA damage as it is one of the predominant forms of free radical-induced lesions of DNA (Phaniendra et al., 2015; Verigos et al., 2020). Its formation in the transcription factor binding sites can modify the binding of these factors and thus change the expression of related genes. In DNA, 8-OHdG leads to the GC to TA transversion mutation (Suzuki and Kamiya, 2016). Due to this, it is known to be mutagenic (Birben et al., 2012). Mitochondrial DNA is more susceptible to ROS damage than nuclear DNA because it is located closer to the site of ROS production. As a result, levels of 8-OHdG are higher in mitochondrial DNA compared to nuclear DNA (Birben et al., 2012).

5-formyl uracil, cytosine glycol, 5,6-dihydrothyronine, 5-hydroxy-6-hydro-cytosine, 5-hydroxy-6-hydro uracil, uracil glycol, and alloxan are also some of the free radical-induced adducts of DNA bases (Phaniendra et al., 2015). Glycolic acid, 2-deoxytetrodialdose, erythrose, 2-deoxypentonic acid lactone, 2-deoxypentose-4-ulose are the important adducts of the sugar moiety in DNA. Oxidization of the guanine base with ROS results in the formation of 8-Hydroxyguanine (8-OHG, the base moiety of 8-OHdG) (Suzuki and Kamiya, 2016). It is an abundant lesion in genomic, mitochondrial, and telomeric DNA and is an essential marker of oxidative damage in DNA.

The RNS, particularly OONO^−^ interacts with guanine on the DNA to produce a nitrative DNA lesion, 8-nitroguanine (8-NO2-G). The produced 8-NO2-G is unstable and can be spontaneously removed, resulting in the formation of an apurinic site (DNA site missing a base analogue). Additionally, during DNA synthesis, adenine can be paired with 8-NO2-G resulting in G-T transversions. As a result, 8-NO2-G is known to be a mutagenic DNA lesion that can contribute to carcinogenesis (Phaniendra et al., 2015). Most of the DNA modifications are implicated in carcinogenesis, aging, neurodegenerative, cardiovascular, and autoimmune diseases (Birben et al., 2012). 8-NO2-G has risen as a marker of RNS-induced nitrative DNA damage (Martinez-Moral and Kannan, 2022).

#### Ribonucleic acid (RNA)

RNA is particularly vulnerable to free radical damage and is more susceptible to oxidative harm than DNA. This increased susceptibility is due to its single-stranded structure, the absence of an effective repair mechanism for oxidized RNA, reduced protection by proteins compared to DNA, and its proximity to mitochondria, the primary site of ROS generation (Vona et al., 2021). Translation of oxidized mRNA can result in the formation of truncated proteins owing to the translation machinery terminating at the oxidized site, or mutated proteins if the entire mRNA has been translated (Liu et al., 2020). As a result, oxidization of RNA can result in altered protein synthesis which can lead to cell degradation and cell death (Liu et al., 2020). This is implicated in various neurological pathologies which will be discussed in the later sections. The attack by RNS on RNA yields the major RNA damage product, 8-hydroxyguanosine or 7,8-dihydro-8-oxo-guanosine (8-oxoG) (Wu and Li, 2008). It appears to be extremely deleterious due to its high mutagenic potential (Wu and Li, 2008). Its levels are elevated in various disease conditions Alzheimer’s disease (AD), Parkinson’s disease (PD), atherosclerosis, hemochromatosis, and myopathies (Phaniendra et al., 2015). 8-oxoG is a reliable marker for oxidative damage of RNA (Guo et al., 2017). Additionally, oxidation of guanosine on the RNA by a nitro (NO2) group yields 8-nitroguanosine (8-NdG) (Kaneko et al., 2008). 8-NdG is an RNA oxidation marker. The quantification techniques for the established DNA and RNA damage markers are enlisted in Table 2.

**TABLE 2:** Nucleic acid damage markers.

Nucleic acid damage markers			
Marker	Sample	Technique	Reference
DNA Damage			
8-Hydroxy-2′-deoxyguanosine (8-OHdG)	Saliva, serum, plasma, tissue, urine	LC-MS, ELISA, HPLC	Chao et al. (2021), Watters et al. (2009)
8-Hydroxyguanine (8-OHG)	Serum, urine, saliva	HPLC HPLC with an ECD	Martinez-Moral and Kannan (2022), Shin et al. (2001), Kawai et al. (2018)
8-Nitroguanine (8-NO2-G)	Peripheral lymphocytes, Urine	HPLC with an ECD HPLC–MS/MS	Martinez-Moral and Kannan (2022), Ohshima et al. (2006)
RNA Damage			
8-hydroxyguanosine (8-oxoG)	Urine	HPLC–MS/MS	Martinez-Moral and Kannan (2022)
8-Nitroguanosine (8-NdG)	Urine	HPLC–MS/MS	Martinez-Moral and Kannan (2022)

Abbreviations: LC-MS, Liquid chromatography mass spectrometry; ELISA, Enzyme-Linked Immunosorbent Assay; HPLC, High-Performance Liquid Chromatography; ECD, electrochemical detector; HPLC–MS/MS, High-Performance Liquid Chromatography with Tandem Mass Spectrometry.

#### Proteins

Oxidant species damage proteins by forming protein-protein cross-linkages, which causes denaturation and leads to the loss of protein functionality, enzyme activity, and functions of receptors and transport proteins (Phaniendra et al., 2015). The free radicals that can attack proteins are O_2_^•^ˉ, ^•^OH, ROO^•^, alkoxyl, and hydroperoxyl, while the non-radical species are H_2_O_2_, O_3_, HOCl, ^1^O_2_, and OONO^−^ (Phaniendra et al., 2015). Following are the various reactions that proteins undergo with oxidant species.

#### Carbonylation

Oxidative damage to the amino acids, lysine, proline, threonine, and arginine yields carbonyl derivatives via protein carbonylation (Phaniendra et al., 2015; Kehm et al., 2021). This reaction results in a stable modification caused by ROS through three pathways: direct oxidation of protein-bound amino acids, oxidative cleavage of the protein backbone, and the incorporation of carbonyls from glycoxidation or lipoxidation (with MDA and 4-HNE reacting with amino groups in proteins) (Kehm et al., 2021). Aminoadipic acid is formed via the ^•^OH mediated abstraction of the hydrogen in lysine (Kehm et al., 2021). Glutamic semialdehyde is formed via the abstraction of a proton from arginine or proline, followed by carbon radical oxidization (Kehm et al., 2021). These instances represent direct oxidation of amino acids, accounting for approximately 60% of the total protein carbonylation observed in the liver (Kehm et al., 2021).

In the process of oxidative cleavage of the protein backbone, the cleavage begins with the formation of alkoxyl radicals mediated by O_2_^•^ˉ at the α-carbon adjacent to a peptide bond. The resulting fragmentation caused by the alkoxyl radical occurs either through the diamide pathway (involving homolytic cleavage of the carbon-carbon bond) or the α-amidation pathway (involving homolytic cleavage of the carbon-nitrogen bond) (Kehm et al., 2021). The former pathway yields diamide and isocyanate as end products, while ketoacyl derivatives and amides are produced in the latter pathway. Glycoxidation-induced carbonylation will be discussed later. The presence of carbonyl groups in proteins serves as an indicator of ROS-mediated protein oxidation. Elevated levels of these carbonyl groups have been linked to various pathologies, including AD, PD, muscular dystrophy, cataract formation, rheumatoid arthritis, diabetes, atherosclerosis, respiratory distress syndrome, and aging. Protein carbonyl content is the most used marker of protein oxidation (Dalle-Donne et al., 2003). It is advantageous to quantify protein-bound carbonyl owing to its frequent occurrence in the body. relatively early formation and the relative stability of oxidised protein moieties (Dalle-Donne et al., 2003). They circulate in the body for longer periods as compared to other parameters of oxidative stress, such as glutathione disulfide (GSSG) or MDA (Dalle-Donne et al., 2003). Lipid peroxidation products are degraded within minutes while cells take hours to days to degrade oxidised proteins (Dalle-Donne et al., 2003).

#### Oxidation of sulfur-containing amino acids

Aminothiol proteins such as cysteine and GSH are highly susceptible to oxidation via alterations of reactive aminothiol residues (Patel et al., 2016). Aminothiols can be measured in serum or plasma to assess the oxidant burden (Patel et al., 2016). Of these aminothiols, cysteine extracellularly accounts for the major aminothiol pool that reacts readily with oxidants. Under enzymatic or non-enzymatic conditions, the thiol group (-SH) in cysteine’s side chain gets oxidized resulting in the formation of a disulfide bond to give cystine (Kehm et al., 2021). Overoxidation of cystine can lead to the oxidation of cysteine sulfenic acid to cysteine sulfinic and finally sulfonic acid (Kehm et al., 2021). Several enzymes can control and reverse the formation and cleavage of disulfide bonds. Therefore, the oxidation of cysteine residues is reversible, except for sulfinic and sulfonic acids (Marrocco et al., 2017a). Owing to cysteine sulfenic being an intermediate, it is not studied as a marker of oxidative stress. Although sulfenic acids are often unstable and reactive, studying this modification may represent the initial product of two-electron oxidants with the thiolate anion, therefore serving as a marker for oxidant-sensitive cysteine residues (Paulsen and Carroll, 2013). Cysteine and its oxidized form, cystine can give the oxidized potential in the body (Kehm et al., 2021). However, owing to cysteine’s instability and high reactivity to be reduced by other thiols, it does not pose as potentially reliable marker of oxidative stress (Paulsen and Carroll, 2013). As a result, cystine appears to be a better marker of oxidative stress.

Methionine is another sulfur-containing amino acid which is highly susceptible to oxidation by ROS (Phaniendra et al., 2015). It can be reversibly oxidized to methionine sulfoxide and irreversibly oxidized to methionine sulfone. Methionine sulfoxide reductases reduce methionine sulfoxide back to methionine. However, they do not target methionine sulfone which is a stable modification (Kehm et al., 2021). As the major oxidation product of protein-bound methionine is methionine sulfoxide, and methionine sulfone, is produced later to much lesser extent, methionine sulfoxide is considered a marker of protein damage by oxidative stress (Ghesquière et al., 2011). Among most thiol oxidized products methionine sulfoxide shows higher stability and is used as an oxidative damage marker.

#### Oxidation of aromatic moieties

Aromatic components within amino acids are preferred targets for protein oxidation (Kehm et al., 2021). Among them, the amino acid tyrosine is particularly susceptible to oxidation. Its phenolic side-chain readily undergoes oxidation, facilitated by the stabilization of the intermediate tyrosyl radical through mesomeric delocalization of the unpaired electron. This tyrosyl radical can then interact with another tyrosyl radical, resulting in the formation of a protein crosslink known as dityrosine (Heinecke et al., 1993). This reaction can be facilitated by oxidative species such as ^•^OH and nitrative species OONO^−^ and nitrosoperoxycarbonate (DiMarco and Giulivi, 2007). Consequently, the presence of dityrosine serves as an indicator of oxidative or nitrative stress (DiMarco and Giulivi, 2007).

ROS and RNS specifically target aromatic amino acid residues, resulting in the formation of dityrosine-containing crosslinks known as Advanced Oxidation Protein Products (AOPP). The levels of AOPP in the body are used as a marker for oxidative stress (Villaverde et al., 2019). 3-nitrotyrosine is a permanent modification formed when tyrosine is nitrated by ^•^NO_2_ attacking the ortho-position of the aromatic ring (Bartesaghi and Radi, 2018). Therefore, nitrotyrosine serves as a biomarker for endogenous OONO^−^ activity and, more broadly, for nitrative stress (Ahsan, 2013). The oxidation of phenylalanine residues by ^•^OH produces abnormal isomers such as ortho- and meta-tyrosine (Kehm et al., 2021). Additionally, ^•^OH oxidizes tryptophan to hydroxytryptophan, which is then cleaved by O_2_ to produce N-formyl kynurenine. A metal-catalyzed reaction of histidine with ^•^OH leads to the formation of 2-oxohistidine (Kehm et al., 2021). 2-oxohistidine has been proposed as a marker of protein oxidation, however, the marker still needs to be studied for its sensitivity and specificity in oxidative stress (Uchida and Kawakishi, 1993).

#### Glycoxidation

Glycation is a protein modification process involving the creation of intermediate Amadori products, which eventually form advanced glycation end products (AGEs) (Kehm et al., 2021). This nucleophilic reaction occurs between amino acid residues and reducing sugars or their reactive degradation products (α-dicarbonyl compounds). Lysine and arginine are particularly susceptible to glycation (Kehm et al., 2021). It is important to note that the formation of AGEs generally does not require oxidative conditions; only certain AGEs are produced through oxidation. These AGEs, formed through both glycation and oxidation, are referred to as “glycoxidation products” (Kehm et al., 2021).

The AGE, carboxymethyl lysine (CML), can be produced through the oxidative degradation of fructoselysine, an Amadori product (Kehm et al., 2021). It can also form from the reaction between the α-dicarbonyl compound glyoxal and lysine through an isomerization process. Although this latter process is non-oxidative, glyoxal itself is predominantly generated by the oxidative degradation of biological molecules such as carbohydrates, lipids, nucleotides, and serine (Kehm et al., 2021). Elevated levels of CML can exert stronger oxidizing potential which may lead to oxidative stress (Gillery, 2006). Therefore, CML levels are used as markers of glycoxidation. A crosslink between lysine and arginine residues yields another important glycoxidation product called pentosidine (Kehm et al., 2021). Although it is found in lower abundance compared to CML, pentosidine is frequently measured glycotoxin in clinical studies and it is important in oxidative stress.

#### Halogenated products

The leukocyte-derived enzyme, EPO generally oxidizes the halide, bromide (Br). 3-bromotyrosine is one of the products formed by the reaction of free and protein-bound tyrosine residues with either HOBr/OBr-. It can also be formed from the reaction with EPO in the presence of H_2_O_2_ and plasma levels of halides (Wu et al., 1999). Halogenated Br products potentially serve as excellent molecular markers to identify sites where EPO promote oxidative damage because there are no other known pathways in the body that result in covalent incorporation of Br into biomolecules. 3-bromotyrosine has risen as an attractive candidate for molecular markers for eosinophil mediated-oxidative damage of proteins by reactive brominating species (Wu et al., 1999).

Stimulated neutrophils generate O_2_^•^ˉ and H_2_O_2_ and release MPO. MPO can catalyse the oxidation of chloride by H_2_O_2_ to give HOCl, which is a strong oxidant that can damage cells (Buss et al., 2003). HOCl reacts with tyrosyl residues in proteins to give 3-chlorotyrosine. This is the only physiologic source of chlorotyrosine which makes it a specific marker for oxidant activity of MPO-containing cells, which include neutrophils and monocytes (Buss et al., 2003).

#### Acrolein

Acrolein is another aldehyde product generated from lipid peroxidation. It is a highly reactive molecule (Il’yasova et al., 2012). Among most lipid peroxidation products, acrolein is by far the strongest electrophile showing high reactivity with nucleophiles, such as the sulfhydryl group of cysteine, imidazole group of histidine, and amino group of lysine (Uchida et al., 1998). Studies state that acrolein was seen to modify lysine and histidine residues of human serum albumin (Gan et al., 1991). The acrolein-lysine adduct has been observed to be the major product of acrolein’s reaction with amino groups (Uchida et al., 1998). The excretion of acrolein-lysine adduct has risen as a biomarker of oxidative status; indicative of damage done to the amino acid (Uchida et al., 1998).

#### Allantoin

Allantoin is the major product of non-enzymatic free-radical oxidation of the antioxidant, UA (Il’yasova et al., 2012). It has emerged as a biomarker for monitoring oxidative status. It is important to note that a variation of UA levels do not correlate with variation in allantoin. This implies that formation of allantoin is independent of UA levels. Hence, allantoin can serve as an effective biomarker of systemic oxidative status (Il’yasova et al., 2012).

The quantification techniques for the protein damage markers have been summarised in Table 3.

**TABLE 3:** Protein damage markers.

Protein damage markers			
Marker	Sample	Technique	Reference
Carbonylation			
Protein carbonylation content	Plasma, serum, tissue, aqueous humor, saliva	Spectrophotometric DNPH assay coupled to protein fractionation by HPLC* ELISA, immunoblot, IHC, cytochemistry	Kehm et al. (2021), Dalle-Donne et al. (2003)
Oxidation of sulfur-containing aromatic amino acids			
Cystine	Serum, plasma	HPLC	Kehm et al. (2021), Patel et al. (2016)
Methionine sulfoxide	Serum, plasma	Western blotting LC-MS techniques	
Oxidation of aromatic amino acids			
Dityrosine	Serum, plasma, urine	LC-MS, spectrophotometric assay spectrofluorimetric assays HPLC–MS/MS	Kehm et al. (2021), Martinez-Moral and Kannan (2022)
Advanced oxidation protein products (AOPP)	Serum, plasma, and saliva	Spectrophotometry*	Kehm et al. (2021), Taylor et al. (2015)
Nitration			
Nitrotyrosine	Serum, plasma, urine	Mass spectroscopy* IHC, ELISA, HPLC, LC-MS	(Kehm et al., 2021; Martinez-Moral and Kannan, 2022)
Glycoxidation			
Carboxymethyl lysine (CML)	Serum, plasma, tissue	ELISA Spectrophotometry IHC, immunoblot HPLC–MS/MS	(Kehm et al., 2021; Martinez-Moral and Kannan, 2022)
Pentosidine	Serum, plasma, tissue	ELISA Spectrophotometry IHC, immunoblot HPLC–MS/MS	(Kehm et al., 2021; Gillery, 2006)
Halogenated products			
3-bromotyrosine	Urine	LC/MS/MS HPLC–MS/MS	(Martinez-Moral and Kannan, 2022; Chen and Chiu, 2008)
3-Chlorotyrosine	Plasma, serum, whole blood, urine	HPLC–MS/MS	(Martinez-Moral and Kannan, 2022; Crow et al., 2016)
**Acrolein**	Urine, tissue	ELISA IHC	(Tamura et al., 2006; Calingasan et al., 1999)
**Allantoin**	Urine	Rimini–Schryver reaction- colorimetric assay* LC-MS/MS HPLC–MS/MS	(Martinez-Moral and Kannan, 2022; Il’yasova et al., 2012)

^*^Indicates the ‘gold standard technique’ for a given marker.

Abbreviations: DNPH, 2,4- dinitrophenylhydrazine; HPLC, High-Performance Liquid Chromatography; ELISA, Enzyme-Linked Immunosorbent Assay; IHC, Immunohistochemistry; LC-MS- liquid chromatography mass spectrometry; HPLC–MS/MS, High-Performance Liquid Chromatography with Tandem Mass Spectrometry; LC-MS/MS, Liquid chromatography electrospray ionization tandem mass spectrometry.

## Antioxidants

The human body is equipped with an antioxidant system that helps combat the effects of oxidants in the body. These antioxidants break radical chain reactions, thereby preventing oxidative stress-related damage. They have heterozygous chemical structures, as their roles require them to work in both hydrophilic and hydrophobic cellular environments (Sharifi-Rad et al., 2020). Antioxidants are generally categorized as enzymatic and non-enzymatic antioxidants. However, from a nutritional point of view, they can also be categorized as endogenous and exogenous antioxidants. Technically, all enzymatic antioxidants are endogenous, as well as some non-enzymatic ones such as thiol antioxidants and coenzyme Q10 (CoQ10) (Sharifi-Rad et al., 2020). On the other hand, exogenous antioxidants are the ones that need to be obtained from the diet since they are not synthesized in eukaryotic cells (Sharifi-Rad et al., 2020). Here, we comprehensively discuss the various enzymatic and non-enzymatic antioxidants.

### Enzymatic antioxidants

In the body, free radicals are quenched by various enzymes. A few of them act directly in scavenging ROS and they are called “primary enzymes,” whereas “secondary enzymes” are the ones that indirectly help in reducing oxidative stress by supporting other endogenous antioxidants (Sharifi-Rad et al., 2020). They have been discussed in detail:

### Primary Enzymes

Primary antioxidant enzymes are the ones that act directly on the main ROS arising from O_2_^•^ˉ and H_2_O_2_ (Sharifi-Rad et al., 2020).

#### Superoxide dismutase (SOD)

**FIGURE 4:**
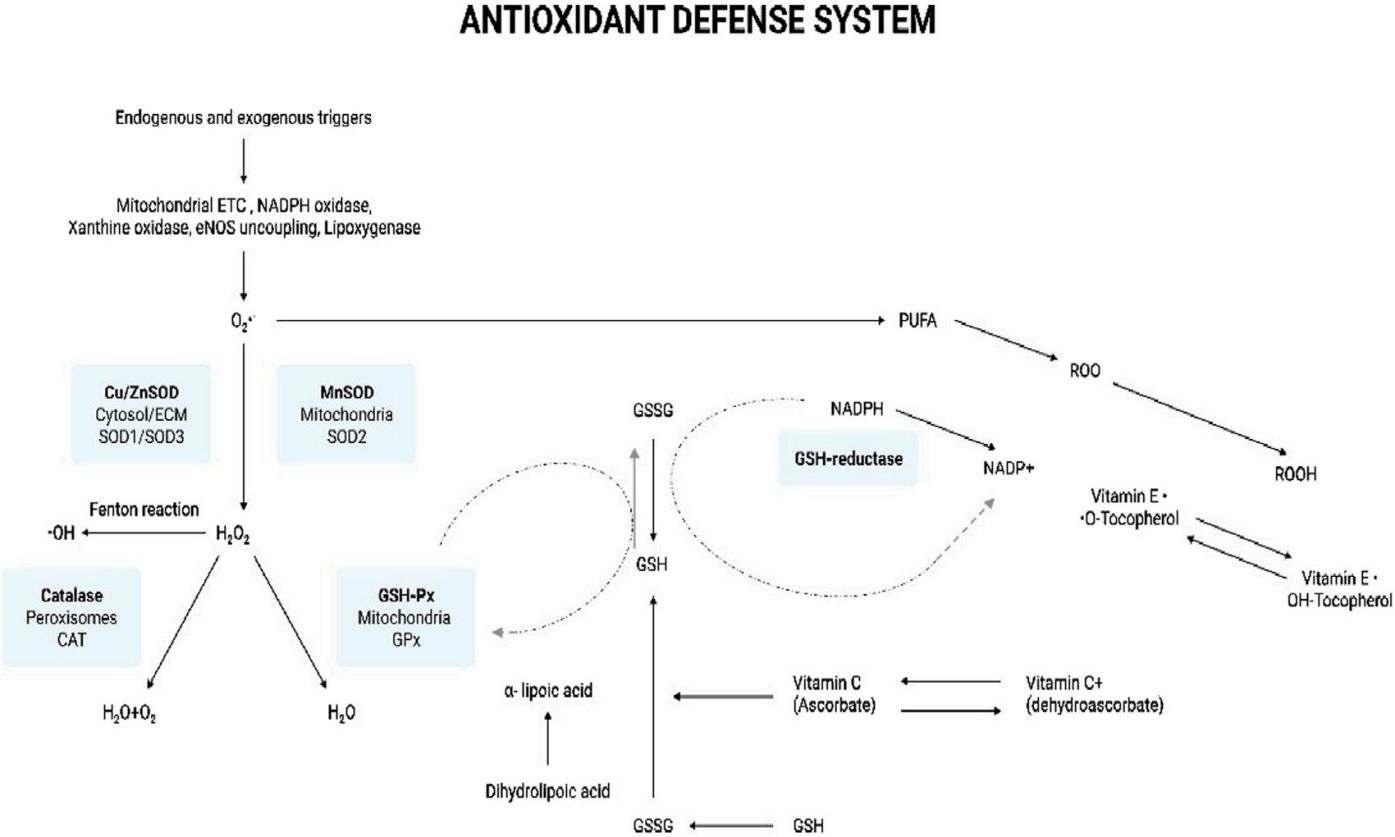
Antioxidant defense system. The illustration indicates the various antioxidants that help combat oxidative stress in the body. Endogenous and exogenous triggers lead to the production of ROS. Mitochondrial ETC, NADPH oxidase, xanthine oxidase, eNOS uncoupling, and lipoxygenase produce O_2_^·^ˉ. The SOD system comprises cytoplasmic Cu/ZnSOD (SOD1), the mitochondrial MnSOD (SOD2), and the extracellular Cu/ZnSOD (SOD3). SOD is the first enzyme to catalyze O_2_^·^ˉ to H_2_O_2_, which, in turn, is reduced to water by catalase and GSH-Px. SOD-derived H_2_O_2_ may contribute to oxidative stress by the production of the ^·^OH radical via the Fenton type reaction. GSH-Px neutralizes oxidant species using GSH as a reducing agent, which results in the formation of the oxidized, GSSG. GSSG is recycled back to GSH through the action of another enzyme called GSH reductase, which uses NADPH as a cofactor. O_2_^·^ˉ can initiate lipid peroxidation by the oxidative degradation of lipids such as PUFAs in cell membranes. This results in the formation of ROO^·^. Vitamin E is an important antioxidant scavenging ROO^·^ and on doing so vitamin E (OH-Tocopherol) gets converted into tocopherol radical (^·^O-Tocopherol). GSH can help regenerate tocopherol by donating electrons to the tocopherol radical, converting it back to its antioxidant form. The important antioxidant, vitamin C (ascorbic acid) can also help regenerate the antioxidant form of OH-Tocopherol from its radical form. Ascorbic acid can be oxidized in the extracellular environment in the presence of metal ions to its less active form, dehydroascorbic acid. Dehydroascorbate can be recycled back to ascorbic acid through various cellular mechanisms facilitated by enzymes and the donation of electrons. The reduced form of OH-Tocopherol indirectly contributes to this recycling process by donating electrons. Additionally, the conversion of GSH to GSSG can also facilitate the regeneration of ascorbic acid from dehydroascorbate. α-Lipoic acid is a very potent antioxidant as it active in both, lipid and aqueous phases. It directly scavenges and neutralizes ROS by donating electrons, thereby reducing oxidative damage. During this process, α-lipoic acid is oxidized to dihydrolipoic acid. Dihydrolipoic itself is a powerful antioxidant and has the ability to recycle and regenerate the other antioxidants, vitamins C and E, back to their active forms. Now, dihydrolipoic can be regenerated to α-lipoic acid by GSH. This regenerative capacity is crucial for maintaining a pool of active antioxidants in the cell and maintaining the cellular defense against oxidative stress. Abbreviations: ETC, Electron transport chain; eNOS - Endothelial nitric oxide synthase; O_2_^·^ˉ, Superoxide anion radical; SOD, Superoxide dismutase; ECM, Extracellular matrix; H_2_O_2_, Hydrogen peroxide; ^·^OH, Hydroxyl radical; GSH-Px, Glutathione peroxidase; H_2_O, Water; O_2_, Oxygen; GSSG, Glutathione disulfide (oxidized form); GSH, Glutathione (reduced form); PUFA, Polyunsaturated fatty acids; ROO^·^, Lipid peroxyl radical; ROS, Reactive oxygen species; Oxidative Stress: Fundamentals and Advances in Quantification Techniques.

SOD the metalloenzyme, primarily catalyses O_2_^•^ˉ dismutation to H_2_O_2_ and O_2_ (Equation 10) (Figure 4) (Sharifi-Rad et al., 2020). In turn, the less harmful H_2_O_2_ can be removed by the other enzymatic antioxidant systems. There are 3 forms of SOD: cytoplasmic Cu/ZnSOD (SOD1), the mitochondrial MnSOD (SOD2), and the extracellular Cu/ZnSOD (SOD3). All 3 forms require catalytic metal (Cu or Mn) for their activation (Fukai and Ushio-Fukai, 2011). The SOD system also competes with ^•^NO for O_2_^•^ˉ. Consequently, SOD also indirectly reduces the formation of another deleterious ROS, OONO^−^ (Equation 11), and increases the ^•^NO biological availability which an essential modulator for endothelial function (Sharifi-Rad et al., 2020). Measurement of the primary antioxidant, SOD is integral in assessing the body’s antioxidant capability.

$$2\;O_{2}^{•}ˉ+2H^{+}\;\to \;H_{2}O_{2}+O_{2}$$ (10)

2 O2•ˉ+N•O → OONO− (11)

#### Catalase (CAT)

H_2_O_2_ produced by SODs or from the action of oxidases, such as xanthine oxidase, is reduced to H_2_O by CAT and GPx (Figure 4). CAT is primarily located in the peroxisomes. It is seen to have the highest activity in liver and red blood cells (Sharifi-Rad et al., 2020). The enzyme exists as a tetramer composed of four identical monomers, each containing a heme group at the active site. CAT neutralizes and maintains an optimum level of H_2_O_2_ in the cell. It breaks down H_2_O_2_ into one molecule of O_2_ and two molecules of H_2_O in a two-step reaction (Alfonso-Prieto et al., 2009). A peroxidase-like compound I intermediate, CpdI is formed at the end of the first step. CpdI is converted back to CAT after a reaction with the second H_2_O_2_ molecule (Equations 12, 13). Recent studies have been indicating the CAT might also help in scavenge OONO^−^ (Gebicka and Didik, 2009). Assessing CAT levels can be indicative of the antioxidant status of the body.

$$\text{CAT }\left({\text{Fe}}^{3+}\right)+H_{2}O_{2}\;\to \;{\text{CpdI}}^{+}\;\left({\text{Fe}}^{4+}=0\right)+H_{2}O$$ (12)

$${\text{CpdI}}^{+}\;\left({\text{Fe}}^{4+}=0\right)+H_{2}O_{2}\;\to \text{ CAT }\left({\text{Fe}}^{3+}\right)+H_{2}O+O_{2}$$ (13)

#### Glutathione peroxidase (GPx)

The GPx enzyme is a selenium-dependent oxidoreductase which is responsible for the reduction of H_2_O_2_ and LOOHs (Birben et al., 2012; Sharifi-Rad et al., 2020). It uses H_2_O_2_ or organic ROO^•^ as the oxidant, and the tripeptide GSH as the electron donor in a general class I peroxidase catalytic cycle (Equations 14, 15) (Figure 4). The enzyme activity depends on the micronutrient cofactor, selenium. For this reason, GPx is often referred to as a selenocysteine peroxidase (Ighodaro and Akinloye, 2017). The GPx family comprises eight isoenzymes (GPx1-8). GPx1 to 4 incorporate selenocysteine which is a non-standard amino acid, where the sulfur atom of cysteine is replaced by selenium. GPx6 contains selenium only in humans, which is not the case with rodents. GPx5, 7, and 8 do not have selenium and instead have a “normal” cysteine (Cardoso et al., 2017).

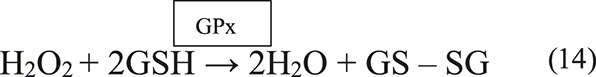




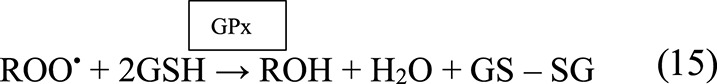




Among all isoforms, GPx1 is the most abundant and is present in virtually all cells. GPx2 is found in the gastrointestinal tract, predominantly in the intestine, while GPx3 is primarily found in the kidney followed by its presence in extracellular fluids as a glycoprotein (Ighodaro and Akinloye, 2017). Although most forms of GPx are tetrameric, GPx4 is a monomer and regarded as is phospholipid hydroperoxide. This is because GPx4 is the only GPx enzyme that breaks down phospholipid hydroperoxides (Ighodaro and Akinloye, 2017). GPx5 is limited to the epididymis of the male reproductive tract in mammals and is regulated by androgens while GPx6 is restricted to embryos and adult olfactory epithelium (Mbemba et al., 2019). GPx7 and GPx8 are present in the endoplasmic reticulum (Mbemba et al., 2019). Quantification of GPx levels can indicate the body’s antioxidant capacity.

GPx’s function is also coupled with the action of the enzyme, glutathione reductase (GR). GPx neutralizes H_2_O_2_ using GSH as a reducing agent. This results in the oxidation of GSH to GSSG. The flavoprotein enzyme, GR, regenerates GSH from its oxidized form, with NADPH as a source of reducing power (Figure 4). Therefore, the action of GR is crucial for enabling GPx’s antioxidant function. Quantification of GR levels is clinically significant as it indicates the level of GR present which helps maintaining the antioxidant pool (Zuzak et al., 2017).

### Secondary enzymes

In addition to the primary enzymes discussed earlier, the degradation of H_2_O_2_ is facilitated by a group of thiol-containing enzymes, which include the thioredoxin system comprising thioredoxins (TRX) and thioredoxin reductases (TRR), thioredoxin peroxidases (PRX), and glutaredoxins (GRX).

#### Thioredoxin system

The thioredoxin system comprises TRX, TRR, and NADPH. It is a major disulfide reductase system which are critical for defense against oxidative stress (Lu and Holmgren, 2014a). The small proteins, TRXs that are thiol antioxidants interact directly with reactive species like H_2_O_2_, ^•^OH, and OONO^−^, and effectively convert them into less harmful molecules. Within cells, there are two primary forms of thioredoxin: one is the cytosolic and nuclear variant called thioredoxin-1 (TRX1), and the other is the mitochondrial isoform known as thioredoxin-2 (TRX2) (Rubartelli et al., 1995). TRXs undergo oxidation while scavenging for oxidants but are subsequently restored to their active, reduced state by TRRs. TRRs are enzymes that utilize NADPH as a cofactor to transfer electrons to the oxidized thioredoxin, converting it back to its reduced and active form, which can then continue its role in maintaining the redox balance within the cell (Tonissen and Di Trapani, 2009). It has been stated that mammalian TRR has three different isoenzymes, cytosolic TRXR1, mitochondrial TRXR2 and TRXR3 (Warner et al., 2004). The TRX system is present in various cellular compartments, allowing it to maintain redox balance and shield the cell against oxidative stress (Lu and Holmgren, 2014a). The TRX protein can be used as a marker, with its increased levels indicative of oxidative stress. The upregulation of TRX is a protective response to counteract the damaging effects of oxidative stress (Oraby and Rabie, 2020; Yokoi et al., 2020).

#### Thioredoxin peroxidases (PRX)

PRX, comprise a large family of thiol-dependent peroxidases that catalyse the reduction of H_2_O_2_, alkyl hydroperoxides, and OONO^−^ (Rhee and Kil, 2017). PRX is among the most abundant proteins in erythrocytes. They catalyse the reduction of H_2_O_2_ or other peroxides, using electrons provided by thioredoxins. In this process, the PRX themselves undergo oxidation and become a disulfide, which is later reduced back to their active form by TRRs. Six PRX isoforms are present in humans – PRDX1, PRDX2, PRDX3, PRDX4, PRDX5, PRDX6 (Ruszkiewicz and Albrecht, 2015). Unlike the other PRDX isoforms that are present in various cellular compartments such as the cytoplasm, mitochondria, and endoplasmic reticulum, PRDX5 is specifically localized in the peroxisomes. Accumulation of oxidized PRX indicates disrupted cellular redox homeostasis, with intermolecular disulfide and hyperoxidized forms accumulating under increased oxidative stress, serving as markers of cellular damage caused by ROS, and compromised redox balance (Lu and Holmgren, 2014b).

#### Glutaredoxins (GRX)

GRX are a family of small redox-regulating proteins that facilitate the reduction of disulfide bonds in target proteins, like thioredoxins. They use GSH as a cofactor in their redox reactions. GRX play a crucial role in cellular defense against oxidative stress and in the repair of damaged proteins. The two most studied human GRXs are the dithiol isoforms GRX1, which mainly exists in the cytosol, and GRX2, which is located in the mitochondria, cytosol or nucleus depending on gene splicing (Jacquot and Zaffagnini, 2019). GRX can be a useful marker for assessing the degree of oxidative stress.

Together, these secondary enzymes comprising thioredoxin-based systems and GRXs contribute to the effective degradation of H_2_O_2_ as well as other oxidative species and help maintain cellular redox homeostasis, thus protecting cells from oxidative damage (Jacquot and Zaffagnini, 2019).

### Non-enzymatic antioxidants

#### Endogenous non-enzymatic antioxidants

##### Glutathione (GSH)

GSH is a tripeptide composed of three amino acids: cysteine, glutamic acid, and glycine. It is the most abundant thiol antioxidant and is present in cytosol, nuclei, and mitochondria. It serves as the major soluble antioxidant in these cell compartments, playing crucial protective roles against oxidative/nitrative stress. It possesses the ability to directly scavenge ^•^OH and ^1^O_2_, bolstering its effectiveness as an antioxidant (Birk et al., 2013). In the body, GSH exists in two isoforms: the reduced form known as GSH and the oxidized form known as GSSG. GSSG is produced when GSH reacts with oxidizing agents such as H_2_O_2_ or free radicals. The antioxidant capacity of thiol compounds, like GSH, is attributed to the presence of a sulfur atom, which readily accommodates the loss of a single electron during free radical neutralization (Shin et al., 2001). Monitoring the levels of total GSH (GSH + 2 GSSG + protein-bound GSH) and the GSH:GSSG ratio serves as reliable indicators of oxidative stress (Bharath et al., 2002). A depletion in these levels and a decreased GSH:GSSG ratio highlight the presence of oxidative stress, signifying the importance of GSH’s role in maintaining cellular redox balance (Warner et al., 2004).

##### Uric acid (UA)

UA is a weak organic acid and the end-product of purine nucleotides degradation. It is an integral part of the body’s antioxidant system. In the extracellular fluid, at a physiological pH of 7.4, UA mainly exists in the ionized form of urate, while in the urine, which is usually acidic, the un-ionized UA form predominates. UA contributes to over half of the blood plasma’s antioxidant capacity (Bowman et al., 2010). It acts as an effective antioxidant, scavenging OONO^−^ and other ROS. Additionally, UA may assist in the removal of O_2_^•^ˉ by inhibiting the degradation of SOD. The removal of O_2_^•^ˉ helps prevent its reaction with ^•^NO, thereby blocking the formation of OONO^−^. In this manner, UA aids in reducing oxidative stress and its elevated levels can serve as a biomarker of oxidative stress (Hwu and Lin, 2010).

##### Albumin

Albumin is the most abundant circulating protein in mammals including humans. It is an antioxidant that is capable of scavenging ^•^OH. It exists in three isoforms named as mercaptalbumin (reduced albumin), non-mercaptalbumin-1 and -2 (oxidized albumin), respectively (Taverna et al., 2013). Oxidization of albumin results in the loss of its antioxidant properties to give, oxidized albumin which further contributes to oxidative stress. Increased levels of oxidized albumin can be indicative of oxidative burden in the body (Kawakami et al., 2006). *In vivo* studies suggest that albumin’s redox state shifts to a more oxidized state in response to the severity of the pathological condition in various diseases such as liver diseases and renal failures (Tabata et al., 2021).

##### Bilirubin

Bilirubin is a yellowish-orange pigment and a byproduct of the breakdown of heme, which is found in haemoglobin, myoglobin, and other heme-containing proteins in red blood cells. Bilirubin exists in various isoforms, with bilirubin IXα being the primary isoform *in vivo* (approximately 99%), while isoforms IIα and XIIIα are present in lower proportions (Hatfield and Barclay, 2004). Bilirubin has been identified as a potent antioxidant, shielding lipids from oxidation by effectively scavenging ROO^•^, and ^1^O_2_. Its presence in serum significantly contributes to the overall antioxidant capacity in blood plasma (Ziberna et al., 2016). This is achieved via its actions on quenching newly formed free radicals, preventing chain reactions that lead to lipid peroxidation. Elevated levels of bilirubin in the bloodstream indicate enhanced antioxidant actions making it a valuable marker for assessing oxidative stress (Ziberna et al., 2016).

##### Coenzyme Q10 (CoQ10)

CoQ10 is a powerful antioxidant naturally found in mitochondria. It is an important component of the electron transport chain where it shuttles electrons between various enzyme complexes as well as accepts free radicals that have escaped and which could form free radicals (Pallotti et al., 2021). It combats oxidative stress by inhibiting lipid peroxidation caused by H_2_O_2_ (Ernster and Dallner, 1995). It has also shown to protect DNA against H_2_O_2_-induced oxidation (Tomasetti et al., 1999). In biological systems, CoQ10 exists in two redox states: the reduced form (ubiquinol, CoQ10H_2_) and the oxidized form (ubiquinone, CoQ10) (Bhagavan and Chopra, 2006). CoQ10’s antioxidant function is mainly attributed to its reduced ubiquinol form (CoQ10H_2_), which is essential for neutralizing free radicals. The CoQ10H_2_ acts as an electron donor in the cellular environment. When exposed to H_2_O_2_ radicals, CoQ10H_2_ donates electrons to neutralize them, effectively transforming H_2_O_2_ into harmless H_2_O and O_2_ molecules. However, this reduced form needs to be continually regenerated from its oxidized form, ubiquinone (CoQ10). Owing to its antioxidant abilities, CoQ10 levels are used as biomarkers to assess oxidative stress (Kaikkonen et al., 1999).

##### Melatonin

Melatonin is an endogenous hormone derived from tryptophan. It is mainly released from the pineal gland in the dark. Along with regulating functions such as sleep, circadian rhythm, immunity, and reproduction, it is also seen to act as an effective antioxidant (Hacışevki and Baba, 2018). Melatonin can easily cross the blood-brain barrier and can enter circulation where it protects biomolecules against damage caused by free radicals by acting as a direct scavenger to detoxify ROS and RNS (Hacışevki and Baba, 2018). It neutralizes ^•^OH and the OONO^−^ generated within the cells. It also scavenges ^1^O_2_, O_2_^•^ˉ, H_2_O_2_, ^•^NO, and HOCl (Hacışevki and Baba, 2018). Moreover, melatonin and its metabolites can also indirectly reduce oxidative stress by enhancing the activities of antioxidative defense systems via stimulating the expression and function of antioxidant enzymes, as well as GSH (Hacışevki and Baba, 2018). It can also inhibit the activity of NOS, which produces ^•^NO. Therefore, melatonin is seen to play an integral role in the body’s antioxidant defenses (Hacışevki and Baba, 2018).

##### α-Lipoic acid (ALA)

ALA, synthesized in the mitochondria, is a caprylic acid-derived antioxidant. It plays an important role in bioenergetic reactions such as the Krebs cycle. It also plays a crucial role in nutrient breakdown. ALA is a sulfur-containing antioxidant. Unlike most antioxidants, which are active only in the lipid or aqueous phase, ALA is active in both phases. It is a very potent endogenous antioxidant as it acts as a chelating agent for metal ions, a quenching agent for ROS (O_2_^•^ˉ, ^•^OH, and HOCl), and a reducing agent for the oxidized form of GSH and vitamins C and E. The presence of heavy metals in the bloodstream are responsible for oxidative stress. However, ALA being an eminent antioxidant, removes metals from the bloodstream via chelation and prevents oxidative stress. Studies have shown that oxidants can lead to cell death via lysosomal breakage caused due to the involvement of intralysosomal iron which catalyses Fenton reactions. This results in peroxidative damage to lysosomal membranes. ALA protects lysosomes against such oxidative insults by chelating intralysosomal iron and consequently, preventing intralysosomal Fenton reactions. On digestion, ALA is converted to dihydrolipoic acid (DHLA). Like ALA, DHLA is also a strong antioxidant that quenches free radicals in both aqueous and lipid phases (Tripathi et al., 2023; Kurutas, 2016).

### Exogenous non-enzymatic antioxidants

#### Vitamin A

Vitamin A encompasses a group of vital fat-soluble compounds known as retinoids and provitamin A carotenoids, with β-carotene being one of the most prominent examples. These compounds play a crucial role as dietary antioxidants, as they possess the remarkable ability to scavenge and neutralize free radicals directly (Fiedor and Burda, 2014). Specifically, β-carotene, when metabolized *in vivo*, acts as a primary antioxidant by scavenging ^1^O_2_. By preventing the formation of LOOHs through its reaction with ^1^O_2_, β-carotene effectively curtails lipid peroxidation, thus safeguarding cellular structures from oxidative damage. Therefore, vitamin A is an important biomarker with its low levels being indicative of oxidative stress (Fiedor and Burda, 2014).

#### Vitamin C

Vitamin C, or ascorbic acid, is a water-soluble essential nutrient obtained through the diet. It exists in various forms, including ascorbic acid and its oxidized form, dehydroascorbic acid. Vitamin C is a potent reducing agent and an important scavenger of oxidants such as ^•^OH, H_2_O_2_, and ^1^O_2_ (Kojo, 2004). While neutralizing oxidant species, vitamin C is rapidly oxidized to DHA and removed from the blood. However, vitamin C can also act as a pro-oxidant, especially in the presence of transition metal ions like iron or copper. This dual function is vital for maintaining cellular redox balance. Monitoring changes in vitamin C levels in the blood can provide insights into the body’s oxidative stress status (Kojo, 2004).

#### Vitamin E

Vitamin E, a fat-soluble antioxidant, comprises eight different types: α-, β-, γ-, and δ-tocopherol, and α-, β-, γ-, and δ-tocotrienol. Among these, α-tocopherol demonstrates the highest antioxidant activity, effectively transferring hydrogen to various ROS like O_2_^•^ˉ and ROO^•^. Its oxidized form can be restored to its active reduced state with the help of ascorbic acid, which donates electrons to the tocopheroxyl radical, converting it back to its antioxidant form, α-tocopherol (Singh et al., 2005). A decrease in vitamin E levels in urine can serve as an indicator of reduced antioxidant status, indicating a compromised ability to combat oxidative stress and maintain cellular health, given its vital role as a primary fat-soluble antioxidant (Wang and Quinn, 2000).

#### Selenium

Selenium is an essential trace element classified as a micronutrient and plays a vital role in various biological processes. It is a part of the group of antioxidant enzymes known as selenoproteins. Selenium acts as a powerful antioxidant, helping to combat oxidative stress by neutralizing harmful free radicals, thereby protecting cells from damage. It specifically helps in preventing lipid peroxidation of H_2_O_2_. Its incorporation into selenoproteins, such as GPxs and thioredoxin reductases, enables these enzymes to detoxify ROS and maintain redox homeostasis. Its levels are often quantified to assess the body’s antioxidant capacity (Zoidis et al., 2018).

#### Zinc

Zinc is a trace element in the human body. Of its many functions, it plays a crucial role in reducing oxidative stress. As an ion, it helps inhibiting the production ROS and RNS via its structural role in antioxidant proteins and its influence on metallothionein induction (proteins rich in thiol groups that are induced to bind and store zinc). By binding to thiol groups of antioxidant enzymes, zinc shields them from oxidation, demonstrating its direct antioxidant activity (Prasad and Bao, 2019). Additionally, zinc functions as a cofactor for the important primary antioxidant, SOD1. Its deficiency can suppress SOD1 activity, making zinc levels an indirect marker of oxidative stress. Decreased zinc levels in cells are often associated with increased oxidative damage (Prasad and Bao, 2019). Monitoring zinc levels may provide insights into the body’s antioxidant defense system and overall oxidative balance (Prasad and Bao, 2019). However, it's worth noting that more studies need to be conducted in humans to further understand the full extent of zinc’s role as a biomarker of oxidative stress.

#### Polyphenols

Polyphenols are natural compounds present in plants that exhibit antioxidant activities. They are ingested via the consumption of fruits, vegetables, cereals, and beverages containing polyphenols. Fruits such as grapes, apples, pear, cherries, and berries, and beverages such as red wine, tea, or coffee, contain polyphenols. Herbs, spices, chocolates, cereals, and dry legumes are also rich in polyphenols (Hussain et al., 2016). 8000 phenolic compounds have been identified in the plants. Polyphenols can include flavonoids such as flavanols, flavones, isoflavones, anthocyanidins, resveratrol, curcumin, tannins, lignans, and phenolic acids (Hussain et al., 2016). The phenolic compounds and flavonoids are known to interact with ROS/RNS and can terminate their reaction. Polyphenols can react with NOS and may modulate the ^•^NO production. Flavonoids such as quercetin, silibin, and luteolin can inhibit the enzyme xanthine oxidase, which produces free radicals (Hussain et al., 2016). Regular intake of polyphenols can boost the body’s antioxidant capacity.

The quantification techniques for the established endogenous and exogenous antioxidant markers are summarized in Tables 4–7. An imbalance between the body’s antioxidant system and oxidants, favoring the generation of oxidants leads to oxidative stress. The Supplementary Data Sheet S1 summarizes the antioxidant capacity of the body to quench and manage the concentrations of various oxidant species in the body.

**TABLE 4:** Endogenous primary enzymatic antioxidant markers.

Endogenous primary enzymatic antioxidant markers			
Marker	Sample	Technique	Reference
Superoxide dismutase (SOD)	Serum, plasma, erythrocytes, tissues, urine	Phenyltetrazol chloride assay INT assay 4-methoxy-6-nitro assay XTT assay NBT assay	(Margaret et al., 2011; Jaruga et al., 1994; Alfonso-Prieto et al., 2009; Gebicka and Didik, 2009; Sinha, 1972)
Catalase (CAT)	Erythrocytes, Serum, Plasma, Tissues	UV spectrophotometry Iodometry Chemiluminescence Polarimetry Titration	(Sinha, 1972; Goth, 1991)
Glutathione peroxidase (GPx)	Erythrocytes, whole blood, plasma, tissue	Spectrophotometry Ellaman’s reagent CUPRAC reagent O-phthalaldehyde reagent Polarographic GSH analysis ELISA	(Sedaghatfard et al., 2016; Yasser et al., 2021)
Glutathione reductase (GR)	Serum, plasma, saliva	ELISA Goldberg and Spooner enzymatic reaction	(Bakirezer et al., 2019; Rezazadeh et al., 2023; Berzosa et al., 2011)

Abbreviations: INT, 2-(4-idophenyl) 3-(4-nitrophenol)-5-phenyltetrazolium; XTT, 3-{1-[(phenylamino)-carbonyl]-3,4-tetrazolium}-bis (4-methoxy-6-nitro) benzenesulfonic acid; NBT, nitro blue tetrazolium; ELISA, Enzyme-Linked Immunosorbent Assay.

**TABLE 5:** Endogenous secondary enzymatic antioxidant markers.

Endogenous secondary enzymatic antioxidant markers			
Marker	Sample	Technique	Reference
Thioredoxins (TRX)	Serum, urine	ELISA	(Oraby and Rabie, 2020; Yokoi et al., 2020)
Thioredoxin peroxidases (PRX)	Erythrocyte	Western blotting Reverse Phase HPLC	(Poynton and Hampton, 2014; Koike et al., 2022)
Glutaredoxins (GRX)	Serum	Fluorescent GRX activity assay	Levin et al. (2018)

Abbreviations: ELISA, Enzyme-Linked Immunosorbent Assay; HPLC, High-Performance Liquid Chromatography.

**TABLE 6:** Endogenous non-enzymatic antioxidant markers.

Endogenous non-enzymatic antioxidant markers			
Marker	Sample	Technique	Reference
Glutathione (GSH)	Whole blood, Plasma, Serum, Tissues, Urine	Ellman’s reagent assay LC-MS/MS Colorimetry Fluorometry HPLC Spectrophotometry	(Skrzydlewska et al., 2005; Araujo et al., 2008)
Uric acid (UA)	Blood, Urine, Serum	Colorimetry LC-MS-TOF, HPLC	(Liao et al., 2006; Duplancic et al., 2011)
Bilirubin	Plasma, serum, urine, feces	Diazo transfer reaction* HPLC Direct spectrophotometry Transcutaneous methods Chemiluminescence Polarography Fluorometry	(Dohi et al., 2005; Narwal et al., 2021)
Coenzyme Q10 (CoQ10)	Plasma, Tissues, Platelets	HPLC-ECD UV-detector HPLC-MS LC-MS/MS	Lagendijk et al. (1996)

^*I^ndicates the ‘gold standard technique’ for a given marker.

Abbreviations: LC-MS/MS, Liquid chromatography electrospray ionization tandem mass spectrometry; HPLC, High-Performance Liquid Chromatography; LC-MS-TOF, Liquid chromatography time-of-flight mass spectrometry; ECD-electrochemical detector; UV, Ultraviolet; HPLC-MS, High-performance liquid chromatography coupled to mass detection.

**TABLE 7:** Exogenous non-enzymatic antioxidants markers.

Exogenous non-enzymatic antioxidants markers			
Marker	Sample	Technique	Reference
Vitamin A	Serum, Plasma, Tissues	APCI/LC-MS Reversed phase HPLC	(Zhu et al., 2006; Kaplan et al., 1990)
Vitamin C	Blood, Tissues, Urine	Dinitrophenylhydrazine method EC-HPLC UV-HPLC Reversed phase HPLC	(Zannoni et al., 1974; Masato, 1980)
Vitamin E	Whole blood, Plasma, Serum, Urine	LC-MS/MS GC-MS Reversed phase HPLC Fluorimetry	Taibi and Nicotra (2002)
Selenium	Plasma, Serum, Blood, Urine	Graphite-furnace atomic-absorption spectrometry HGAAS MFS ICP-MS	Marrocco et al. (2017b)

Abbreviations: APCI, Atmospheric pressure chemical ionization; LC-MS- liquid chromatography mass spectrometry; HPLC, High-Performance Liquid Chromatography; EC- electrochemical detection; UV- ultraviolet; LC-MS/MS, Liquid chromatography electrospray ionization tandem mass spectrometry; GC-MS, Gas Chromatography-Mass Spectrometry; HGAAS, Hydride-generation atomic absorption spectrometry; MFS- molecular fluorescence spectrometry; ICP-MS - HPLC, coupled to inductively coupled plasma-mass spectrometry.

## Oxidative stress in aging

Aging is defined as an intrinsic, multifactorial, and progressive process characterized by tissue degeneration and progressive loss of organ function, ultimately leading to increased mortality (Liguori et al., 2018; Tan et al., 2018). Of the many theories, the “free radical theory of aging,” also known as the “oxidative stress theory of aging” has been of great interest (Tan et al., 2018). The theory hypothesizes that aging is associated with structural impairment caused due to the accumulation of oxidative damage to crucial macromolecules (lipids, DNA, RNA, and proteins) brought about ROS and RNS (Liguori et al., 2018). The increase in oxidative stress could be brought about by the failure of several defensive mechanisms to respond to the ROS-induced damage, particularly in the mitochondria (Tan et al., 2018).

Aging is associated with structural and functional changes in the mitochondria (Seo et al., 2016), which is accompanied by the alterations of biophysical properties of the membrane including alteration in the electron transport chain complex activities, decreased fluidity, and energy imbalance and mitochondrial failure (Tan et al., 2018). Reduced oxidative phosphorylation results in increased ROS production (Iakovou and Kourti, 2022). This gives rise to impaired cellular homeostasis and mitochondrial function leading to the increased vulnerability to oxidative stress (Tan et al., 2018). Increased ROS can activate the pro-apoptotic protein, p66Shc which further contributes to the production of ROS. This, in turn, promotes the accelerated damage of the mitochondria, leading to apoptosis and finally resulting in the process of aging (Iakovou and Kourti, 2022). Therefore, p66Shc which is responsible for ROS generation and apoptosis induction is regarded as a link between ROS and aging (Iakovou and Kourti, 2022).

NAD+ is an important coenzyme involved in cellular redox reactions. It helps maintain mitochondrial function, redox homeostasis, anti-inflammatory action, and attenuates age-related dysfunctions (Alegre and Pastore, 2023). Enhanced levels of NAD + are known to activate pro-survival pathways (Poljsak and Milisav, 2016). The depletion of NAD + or the NAD+/NADH ratio can influence the formation of ROS by altering the regulation of intracellular ATP production, redox state and metabolic enzymes (Poljsak and Milisav, 2016). With progressing age, NAD+ and nicotinamide mononucleotide (NMN) levels reduce and NADH levels increase. Excessively high NADH levels were reported to lead to reductive stress, a state characterized by the increase in reducing equivalents in the presence of intact systems for oxidation and reduction. This increase in NADH concentration results in ROS formation by NADH- induced iron release from ferritin or the electron transport chain (Poljsak and Milisav, 2016).

It is important to note, that not only the increased production of ROS and RNS but also the decline in the efficiency of antioxidant systems with age leads to oxidative stress (Liguori et al., 2018). A study conducted by Reddy et al. (1998), assessed the levels of lymphocyte free radical generation (O_2_^•^ˉ & H_2_O_2_), DNA damage, and antioxidant enzyme levels (GST, SOD, and CAT) in healthy individuals between 20–80 years (Reddy et al., 1998). They found that O_2_^•^ˉ & H_2_O_2_ progressively increased while the antioxidant enzyme levels showed a gradual decrease from younger to older age (Reddy et al., 1998). The reduction in antioxidant expression can be linked to the age-related decline in Nrf2/ARE activity (Zhang et al., 2015). Nuclear factor erythroid 2-related factor 2 (Nrf2), a critical transcription factor, regulates antioxidant and detoxification enzymes. Under normal conditions, Nrf2 is bound to Kelch-like ECH-associated protein 1 (Keap1) in the cytosol. Oxidative modification of Keap1’s cysteine residues impairs this binding, allowing Nrf2 to dissociate and translocate to the nucleus. There, Nrf2 binds to the antioxidant response element (ARE), promoting the expression of antioxidants such as SOD, CAT, GPx, glutathione S-transferases, sulfiredoxin, and thioredoxin reductase (Zhang et al., 2015; Re et al., 2014). Thus, Nrf2/ARE activity is essential for combating oxidative stress and facilitating cellular repair (Re et al., 2014). Various studies have demonstrated changes in Nrf2/ARE activity associated with aging. This is supported by a reduction in nuclear Nrf2 levels and its binding to the ARE motif in older organisms, potentially leading to lower antioxidant expression (Zhang et al., 2015). Although it is well-established that both basal and inducible (in response to stress) antioxidant levels are regulated by Nrf2/ARE, research has shown that age-related changes in Nrf2/ARE primarily reduce inducible antioxidant levels (Zhang et al., 2015). There is no consensus on whether this hypothesis affects basal antioxidant levels.

Basal levels of antioxidants are influenced by factors such as genetics, diet, medications, disease pathologies, and environmental stressors (Zhang et al., 2015). Considering this, basal antioxidant levels can be altered due to changes in nutrition and hormones with age. Malnutrition in older individuals resulting from poor nutritional habits, loss of appetite, or intestinal malabsorption may lead to deficiencies in trace elements such as Zn^2^+ ions, essential for SOD1 activity or selenium, essential for the synthesis of selenoenzyme GPx, thus weakening the body’s antioxidant system (Kozakiewicz et al., 2019). The age-associated reduction in the secretion of the pineal hormone, melatonin which regulates both, the expression of genes coding for antioxidant enzymes such as SOD, GPx, and GR and directly influences their activities can also be the cause of declining antioxidant capabilities with age (Reddy et al., 1998).

Studies have shown that oxidative stress can induce cellular senescence which is another factor that leads to aging. It is a physiological mechanism that stops cellular proliferation in response to damages that occur during replication (Liguori et al., 2018). Oxidative stress can promote cellular senescence as it causes DNA lesions, accelerates telomere shortening, and activates molecular pathways leading to growth arrest (Iakovou and Kourti, 2022). Senescent cells acquire an irreversible senescence-associated secretory phenotype (SASP). SASP involves the secretion of soluble factors (interleukins, chemokines, and growth factors), degradative enzymes like matrix metalloproteases (MMPs), and insoluble proteins/extracellular matrix components. ROS and RNS can induce cellular senescence by exerting effects on various SASP components (Liguori et al., 2018).

Oxidative stress leading to cellular senescence by affecting SASP components is the pathogenesis of various conditions including cardiovascular diseases, acute and chronic kidney disease, neurodegenerative diseases, macular degeneration, biliary diseases, and cancer (Liguori et al., 2018). Vascular calcification which is a pathophysiological consequence of atherosclerosis can be caused due to SASP-driven osteoblastic trans differentiation of senescent smooth muscle cells (Liguori et al., 2018). In the neurodegenerative condition, AD, brain tissue biopsies were shown to have increased levels of p16, MMP, and IL-6 (Liguori et al., 2018). Oxidative stress is fundamental in age-associated conditions, thereby affecting lifespan and longevity. Increased inflammation is a pervasive feature of aging (Chung et al., 2019). Given the close relationship between oxidative stress, inflammation, and aging, the oxidation-inflammatory theory of aging or ‘oxi-inflamm-aging’ has been hypothesized. The theory believes that aging is the resultant of the loss of homeostasis due to a chronic oxidative stress that affects the regulatory systems, including the nervous, endocrine, and immune systems. This may result in the consequent activation of the immune system giving rise to an inflammatory state. In this manner, chronic oxidative stress and inflammation feed each other forming a continuous vicious cycle, and consequently, increases the age-related morbidity and mortality (Liguori et al., 2018).

## Future prospective

Oxidative stress is an inevitable phenomenon, making it essential to maintain its levels optimally. Quantification techniques can help detect oxidative damage markers and antioxidant markers in various biological samples such as tissue, saliva, serum, plasma, and urine. Convenient collection samples and effective detection of oxidative stress biomarker can enable the effective analysis of oxidative stress. Understanding its fundamentals and accurately quantifying oxidative stress can elucidate the nuanced changes that this phenomenon induces in health and disease states. Furthermore, precise assessment of oxidative stress can facilitate the development of mitigation strategies. Continued research in this domain can lead to personalized interventional and therapeutic approaches aimed at optimizing redox function, minimizing the risk of oxidative stress-mediated conditions, and ultimately promoting longevity.

## Limitations

The study provided an extensive understanding of various aspects of oxidative stress and its measurement techniques. However, it has several limitations that need to be considered. Firstly, the incorporation of studies with diverse methodological quality could contribute to significant heterogeneity, potentially affecting the robustness and coherence of the conclusions. Secondly, the objective to offer a broad overview of oxidative stress might have resulted in insufficient depth and detail regarding specific mechanistic insights, limiting a thorough examination of particular facets of the topic. Lastly, the study exclusively relied on published literature, potentially neglecting unpublished or negative findings, which may have led to a bias in the content presented.

## Conclusion

Oxidative stress is a phenomenon in which excessive oxidant species attack cellular macromolecules such as lipids, nucleic acids, and proteins. Studies have indicated that oxidative stress is an important factor driving the process of aging and it can also be associated with age-related pathologies. This warrants the need to assess and effectively understand mechanisms of oxidative stress in the body along with its reliable quantification. As directly quantifying oxidative stress is not feasible, indirect quantification of oxidative stress by measuring oxidative damage markers (lipid peroxidation, nucleic acid and protein damage markers) and antioxidants (enzymatic and non-enzymatic) can indicate the degree of oxidative stress in the body. Oxidative stress is involved in the mechanism of aging. Managing oxidative stress could delay the expression of SASP factors that leads to cellular senescence, therefore delaying aging.

## Glossary

ROS: Reactive oxygen species
RNS: Reactive nitrogen species
**O**_**2**_^**•**^ **ˉ**: Superoxide anion radical
**H**_**2**_ **O**_**2**_: Hydrogen peroxide
^**•**^ **OH**: Hydroxyl radicals
^**1**^ **O**_**2**_: Singlet oxygen
SOD: Superoxide dismutase
CAT: Catalase
GPx: Glutathione peroxidase
UA: Uric acid
GSH: Glutathione
**ROO** ^**•**^: Peroxyl radical
^**•**^ **NO**: Nitric oxide
^**•**^ **NO**_**2**_: Nitrogen dioxide
HOCl: Hypochlorous acid
HOBr: Hypobromous acid
**O**_**3**_: Ozone
**HNO**_**2**_: Nitrous acid
**NO** ^**+**^: nitrosyl cation
**NO** ^**−**^: Nitroxyl anion
**N**_**2**_ **O**_**3**_: Dinitrogen trioxide
**N**_**2**_ **O**_**4**_: Dinitrogen tetraoxide
**NO**_**2**_^**+**^: Nitronium (nitryl) cation
ROOH: Hydroperoxides
HCOR: Aldehydes
**OONO** ^**−**^: Peroxynitrite
**O**_**2**_: Molecular oxygen
**HO**_**2**_: Hydroperoxyl radical
**HOO** ^**•**^: Per hydroxyl radical
NOS: Nitric oxide synthases
nNOS: Neuronal NOS
eNOS: Endothelial NOS
iNOS: Inducible NOS
**NO**_**2**_^**-**^: Nitrite
**NO**_**3**_^**-**^: Nitrate
**CO**_**2**_: Carbon dioxide
**ONOOCO**_**2**_^**−**^: Peroxo carboxylate
ONOOH: Peroxynitrous acid
**NO**_**2**_^**∙+**^: Nitrosonium cation
HOCl: Hypochlorous acid
HOBr: Hypobromous acid
MPO: Myeloperoxidase
EPO: Eosinophil peroxidase
^**∙**^ **Q** ^**-**^: Semiquinone anion
NOX: NADPH oxidase
UVA: Ultraviolet A
8-oxoGua: 8-Oxo-7,8- dihydroguanine
PFAS: Per- and polyfluoroalkyl substances
GST: Glutathione-S-transferases
GR: Glutathione reductase
BPA: Bisphenol A
BPAG: Bisphenol A glucuronide
BPAS: Bisphenol A sulfate
Dox: Doxorubicin
GSTP1: Glutathione S-transferase pi 1
H₂DCFDA: 2´7´-dichlorofluorescin diacetate
H₂DCF: 2’7’-dichlorofluorescin
DCF: 2’,7’-dichlorofluorescein
DHE: Dihydroethidium
2-OH-E+: 2-hydroxyethidium
E+: Ethidium
cGMP: cyclic guanosine monophosphate
MAPK: Mitogen-activated protein kinase
PI3Ks: Phosphoinositide 3-kinases
PTEN: Phosphatase and TENsin homolog
Nrf2: Nuclear factor erythroid 2-related factor 2
Keap1: Kelch-like ECH-associated protein 1
NQO1: NAD(P)H quinone oxidoreductase 1
Srx1: Sulfiredoxin 1
PUFA: Polyunsaturated fatty acids
**CH**_**2**_: Methylene group
**L** ^**•**^: lipid radical
**LOO** ^**•**^: Lipid peroxyl radical
LA: Linoleic acid
AA: Arachidonic acid
EPA: Eicosapentaenoic acid
DHA: Docosahexaenoic acid
LOOH: Lipid hydroperoxides
H(P)ODEs: Hydro(pero)xyoctadienoates
MDA: Malondialdehyde
4-HNE: 4-Hydroxy-2-nonenal
4-HNE-MA: 4-Hydroxynonenalmercapturic acid
4-ONE: 4-Oxo-2-nonenal
CML: Carboxymethyl lysine
PG: Prostaglandin
IsoPs: Isoprostanes
F2-IsoP: F2-isoprostanes
**8-isoPGF**_**2**_ **α**: 8-isoprostaglandin F_2_α
11-PGF2α: 11-β-prostaglandin F2α
15-PGF2α: 15-prostaglandin F2α
8-OHdG: 8-Hydroxy-2’-deoxyguanosine
8-OHG: 8-Hydroxyguanine
8-NO2-G: 8-nitroguanine
8-oxoG: 8-hydroxyguanosine or 7,8-dihydro-8-oxo-guanosine
AD: Alzheimer’s disease
PD: Parkinson’s disease
8-NdG: 8-nitroguanosine
GSSG: Glutathione disulfide
AOPP: Advanced Oxidation Protein Products
AGEs: Advanced glycation end products
CoQ10: Coenzyme Q10
TRX: Thioredoxins
TRR: Thioredoxin reductases
PRX: Thioredoxin peroxidases
GRX: Glutaredoxins
TRX1: Thioredoxin-1
TRX2: Thioredoxin-2
**CoQ10H**_**2**_: Ubiquinol
ALA: α-Lipoic acid
DHLA: Dihydrolipoic acid
NMN: Nicotinamide mononucleotide
ARE: Antioxidant response element
MMPs: Metalloproteases
SASP: Senescence-associated secretory phenotype
