# Supplementary material for: Oxidative stress: fundamentals and advances in quantification techniques
Source: Front Chem. 2024 Oct 7;12:1470458. doi: 10.3389/fchem.2024.1470458 (PMC11491411; doi:10.3389/fchem.2024.1470458)
Supplement: Supplementary file 1 [file DataSheet1.docx]

**Oxidative Stress: Fundamentals and Advances in Quantification Techniques**

Hari Krishnan Krishnamurthy^1^*, Michelle Pereira,^2^ Imbaasree R,^2^ Vasanth Jayaraman,^1^ Karthik Krishna,^1^ Tianhao Wang,^1^ Kang Bei,^1^ John J. Rajasekaran^1^.

1 Vibrant Sciences LLC., Santa Clara, CA, United States of America

2 Vibrant America LLC., Santa Clara, CA, United States of America

**Supplementary Material**

OXIDATIVE DAMAGE MARKERS

1. **Quantification of lipid peroxidation markers**
2. Lipid hydroperoxides (LOOHs)

LOOHs are commonly used as biomarkers of oxidative stress-associated pathological conditions. Generally, the chemical stability of LOOHs is concerning as LOOHs are the primary oxidation products that can immediately be transformed into secondary products [42]. However, LOOHs can be measured using various techniques: chemiluminescence-based High-Performance Liquid Chromatography (HPLC) detection can be used to detect in tissue samples while the Iodometric Assay detects them in serum [43,44]. Ferrous Oxidation of Xylenol (FOX) assay can be used to detect LOOHs in plasma and serum lipoproteins [44].

1. Malonaldehyde (MDA)

Circulating MDA can be detected either free (unconjugated) or conjugated forms. MDA bound to various biological molecules such as proteins, nucleic acids, lipoproteins, and soluble amino acids accounts for the conjugated form [45, 46]. The sum of the two forms gives the total MDA [46]. MDA can be measured in serum, plasma, urine, CSF, erythrocytes, and saliva [34]. As the collection of urine samples are easy, convenient, and non-invasive, urinary MDA is now considered as a biomarker of systemic oxidative stress [46]. The thiobarbituric acid reactive substances (TBARS) assay is the gold-standard assay used to measure MDA levels owing to its simplicity and low costs. However, the assay has been criticized for being non-specific for MDA [34]. However, modifications including HPLC/GC (Gas Chromatography-Mass Spectrometry)-based isolation of (MDA)-TBAR product and spectrophotometric detection can be used to quantify MDA [34].

1. 4-hydroxy-2-nonenal (4-HNE)

4-HNE is a good marker of lipid peroxidation and can be measured in serum, plasma, urine, CSF, and tissue [47, 34]. The golden-standard method used for the measurement of 4-HNE is based on the formation of an oxime derivative using O-(2,3,4,5,6-pentafluorobenzyl) hydroxylamine (PFBHA) followed by silylation of the hydroxyl group prior to GC-MS analysis [47]. This method is sensitive to the physiological concentrations of 4HNE in urine samples [47]. Martinez-Moral et al and colleagues were able to quantify urinary 4HNE levels using High-Performance Liquid Chromatography with Tandem Mass (HPLC–MS/MS) [48]. Qualitative and semiquantitative immunological methods, such as Enzyme-Linked Immunosorbent Assay (ELISA)and Immunohistochemistry (IHC) can also be used to quantify 4-HNE in biological samples [34].

1. 4-Hydroxynonenalmercapturic acid (4-HNE-MA)

4-HNE produced from lipid peroxidation, can form an adduct with the antioxidant GSH, producing 4-hydroxynonenal glutathione (4-HNE-GSH). 4-HNE-GSH gets further metabolized in the kidney to form, 4-Hydroxynonenalmercapturic acid (4-HNE-MA). 4-HNE-MA then gets excreted via urine. As a metabolite of 4-HNE, urinary levels of 4-HNE-MA act as indicators of lipid peroxidation in the body. A study quantified 4-HNE-MA in urine using HPLC–MS/MS [48].

1. 4-oxo-2-nonenal (4-ONE)

4-oxo-2-nonenal (4-ONE) is a product of lipid peroxidation. Like other lipid peroxidation markers, it is also a reactive HCOR and its free levels are unstable. It is a marker of oxidative stress. Its metabolism is like 4-HNE However, it is a less studied oxidative stress biomarker as compared 4-HNE [49]. Nevertheless, it can be detected in urine using isotope-dilution mass spectrometry [50].

1. F2-isoprostanes

Circulating levels of levels of 8-isoprostaglandin F_2_α (8-isoPGF2α) can be measured in plasma and urine. However, for oxidative stress, the urinary assessment of 8-isoPGF2α is preferred over its plasma levels, due to the ex vivo, artefactual formation of IsoPs resulting from auto-oxidation of lipids in plasma [45]. Urinary 8-isoPGF2α can be accessed via the gold standard technique ‘GC-MS’ [45,47]. It can also be measured by competitive ELISA kits using antibodies [45]. As 8-isoPGF2α is a relatively minor F2-IsoPs in urine, cross-reactivity of the antibodies needs to be checked carefully [45]. It can also be measured using HPLC–MS/MS [48]. Additionally, the other F2-IsoPs products, 11-β-prostaglandin F2α (11-PGF2α) and 15-prostaglandin F2α (15-PGF2α) as well as the isomer of 8-isoPGF2α, 8-Iso-15(R)-Prostaglandin F2α whose levels are increased during oxidative stress, can also be measured in urine using HPLC–MS/MS [48]. The techniques for the mentioned F2-IsoP metabolite have been individually stated in table 1.

**II. Quantification of Nucleic acid damage markers**

*Deoxyribonucleic Acid (DNA)*

1. 8-Hydroxy-2’-deoxyguanosine (8-OHdG)

The half-life of the oxidative DNA damage marker, 8-OHdG is longer than other oxidized products, and it can be easily detected in clinical practice [53]. It can effectively be measured in saliva, serum, plasma, tissue, and urine [56]. The techniques used for quantification are LC-MS, ELISA, and HPLC techniques [57].

1. 8-Hydroxyguanine (8-OHG)

8-OHG is a marker of oxidative damage in DNA [58]. 8-OHG can be measured in serum and urine using HPLC [48, 59]. It can also be measured in saliva using an HPLC system equipped with an electrochemical detector (ECD) [60].

1. 8-nitroguanine (8-NO2-G)

The RNS-induced nitrative DNA damage marker, 8-NO2-G can be detected in peripheral lymphocytes using HPLC with an ECD [61]. Owing to its convenience, urinary 8-NO2-G has risen as a marker of RNS-induced nitrative DNA damage [62]. 8-NO2-G can be measured in urine using HPLC–MS/MS [48].

Ribonucleic acid (RNA)

1. 8-hydroxyguanosine (8-oxoG)

8-oxoG is stable and is relatively easily formed which makes it a good marker for oxidative damage of RNA [53]. Urinary levels of 8-oxoG can be measured by HPLC–MS/MS [48].

1. 8-nitroguanosine (8-NdG)

The RNA oxidation marker, 8-NdG’s production is enhanced under inflammatory conditions and is known to be a mutagenic RNA lesion [65]. 8-HdG can be measured in serum and urine using HPLC [48,58].

**III. Quantification of Protein damage markers**

1. Protein Carbonylation

Protein carbonylation content can be effectively determined in plasma, serum, tissue samples, aqueous humor and saliva [34]. Although saliva poses a convenient, non-invasive, pain- and infection-free means of sample collection; the sample could get affected by oral hygiene [34]. Nevertheless, urinary quantification appears to be an alternate non-invasive marker of protein carbonylation content. The most common method used to quantify protein carbonylation is the reaction with 2,4- dinitrophenylhydrazine (DNPH) which leads to the formation of a stable 2,4-dinitrophenyl (DNP) hydrazone product; this reaction was pioneered by Levine et al. [66]. The stable DNP adduct can be measured by a spectrophotometric assay, which can be coupled to protein fractionation by HPLC [66]. Using specific anti-DNP antibodies, protein carbonyls can be measured via ELISA, immunoblot (western blot), IHC and cytochemistry techniques [66].

1. Oxidation of sulfur-containing amino acids

Of the aminothiols, cysteine extracellularly accounts for the major aminothiol pool that reacts readily with oxidants to form, oxidized disulphide cystine. Therefore, cysteine and its oxidized form, cystine can give the oxidized potential in the body [34]. However, as cysteine is instability and can be readily reduced by other thiols, it is not a reliable marker of oxidative stress [69]. Cystine appears to be a better marker of oxidative stress. Cystine can be measured in plasma or serum using HPLC [34,67].

Methionine sulfoxide is stable in comparison to the other oxidized thiol products. It can be quantified in serum or plasma using via immunoblotting (western blotting) or LC-MS techniques [34]. Despite it being suitable for laboratorial analysis, it is not actively assessed as an oxidative stress biomarker in clinical settings [34]. This could be due to methionine sulfoxide failing to give the true value of oxidized protein, as it can be converted to methionine via redox signalling and regulatory mechanisms in cells.

1. Oxidation of aromatic moieties

Dityrosine is a reliable biomarker owing to its chemical stability (unreactive to alterations in oxygen and/or pH), and its non-involvement in de novo synthesis of proteins [72]. It can be quantified in serum, plasma, and urine using LC-MS, spectrophotometric or spectrofluorimetric assays [34]. Urinary levels of dityrosine can be conveniently measured by HPLC–MS/MS [48].

Advanced Oxidation Protein Products (AOPP) can be measured in serum, plasma, and saliva using spectrometric assays based on the oxidation reaction of iodide to iodate [34]. However, this technique was prone to sample precipitation and consequent poor reproducibility [73]. Taylor et al., 2015 suggested a modified assay by determining total iodide ion oxidizing capacity of plasma and eliminating the effect of sample precipitation. This improved accuracy and reproducibility of the assay, allowing the researchers to effectively measure AOPP in the plasma of diabetic patients [73].

Mass spectroscopy is considered the gold standard technique for detecting nitrotyrosine in various biological samples such as plasma, serum, and tissue [34]. Nevertheless, the biomarker can also be detected using IHC, ELISA, HPLC or the combination of LC with MS/MS [34]. Owing to the standardization, easy sample preparation, and high sample throughput, commercially available ELISAs (indirect) are preferentially used for nitrotyrosine quantification in clinical studies [34]. Nitrotyrosine can be measured in urine using HPLC–MS/MS which can pose as a convenient and non-invasive means for assessing nitrative stress [48].

1. Glycoxidation

Carboxymethyl lysine (CML) can be measured in serum, plasma, urine, and tissue samples using spectrophotometry, IHC, immunoblot, and ELISA techniques, with ELISA being the most commonly used technique [34]. Urinary levels of CML can be a convenient means to monitor the degree of oxidative stress in the body system and they can be measured using HPLC–MS/MS [48].

Pentosidine can be detected in serum, plasma, urine, and tissue samples using spectrophotometry, IHC, immunoblot, and ELISA techniques. ELISA has been most commonly used technique [34]. As urine is a non-invasive and convenient to collect, pentosidine can be quantified in urine using HPLC [78].

1. Halogenated products

3-bromotyrosine is measured in urine using isotope dilution liquid chromatography electrospray ionization tandem mass spectrometry (LC-MS/MS) or HPLC–MS/MS [48,79].

3-chlorotyrosine can be quantified in plasma, serum, or whole blood via HPLC–MS/MS [80]. A more convenient and non-invasive means to quantify the biomarker would be assess its urinary using HPLC–MS/MS [48].

1. Acrolein

The acrolein-lysine adduct can be quantified using competitive ELISA [83]. High cross-reactivity of the assay and the fact of acrolein-lysine not being the product and possibly undergoing further thiolation are seeming as limiting factors that are keeping protein-bound acrolein from being an effective marker of oxidative stress [49]. Acrolein-protein adducts can also be quantified in tissues using IHC [84].

1. Allantoin

Allantoin has been measured by the colorimetric assay based on the Rimini–Schryver reaction but improved methods such as LC-MS/MS have been able to quantify the biomarker with minimal sample preparation [49]. The urinary concentration of allantoin is not affected by freeze/thaw cycles, sample preparation, or by storage at room temperature for up to 6 days, 12 days at 4°C, or, at minimum, 15 weeks at −70°C [49]. It can also conveniently be measured in urine using HPLC–MS/MS [48].

ANTIOXIDANT MARKERS

1. **Endogenous enzymatic antioxidants**

*Primary enzymes*

1. Superoxide dismutase (SOD)

SOD can be detected in various biological samples, such as serum, plasma and erythrocytes, tissue homogenates, cell lysates, and urine. Among these, SOD levels are commonly measured in erythrocytes to assess the systemic antioxidant status. Both activity assay and activity gel can be used to measure SOD levels. These assays provide a measure of the amount of SOD present in the sample [87]. Most procedures for determining SOD concentration are performed by indirect assessment methods, usually by addition to the erythrocyte sample, for instance, 2-(4-iodophenyl)-3-(4-nitrophenol)-5-phenyltetrazol chloride or by using tetrazolium salts such as 2-(4-idophenyl) 3-(4-nitrophenol)-5-phenyltetrazolium (INT), 3-{1-[(phenylamino)-carbonyl]-3,4-tetrazolium}-bis (4-methoxy-6-nitro)benzenesulfonic acid (XTT), or nitro blue tetrazolium (NBT) [88].

1. Catalase (CAT)

CAT can be quantified in various biological samples, such as erythrocytes, serum, plasma, and tissue homogenates. UV spectrophotometric method is commonly used to assess CAT levels as it is simple, relatively quick, and cost-effective [91]. However, high H_2_O_2_ levels can inhibit CAT activity which may cause variations in the quantification of CAT levels using this method. Other methods of measuring CAT levels have been developed, including those involving iodometry, chemiluminescence, polarimetry, and monitoring the production of oxygen via an oxygen electrode or a low-flow gas meter [92]. However, these methods are often time-consuming and may not be suitable for routine clinical use due to their complexity and resource requirements. Despite its limitation, the UV spectrophotometric method remains the most commonly used technique for measuring CAT levels due to its widespread availability and ease of use [92].

1. Glutathione peroxidase (GPx)

GPx can be measured in different biological samples, such as erythrocytes, whole blood, plasma, and tissue homogenates. Un-clotted EDTA or heparin whole blood samples are commonly used as GPx values have been linked to hemoglobin. GPx activity refers to the neutralizing potential of GPx. GPx activity can be accessed via conventional methods like spectrophotometry or colorimetry. Spectrophotometry measures GPx activity by linking its reaction with GSH reductase, measuring the conversion of NADPH to NADP. Another way is by measuring residual GSH content by using Ellman's reagent. Some methods use the CUPRAC reagent for spectrophotometric detection, while others use o-phthalaldehyde as a fluorescent reagent [96]. A method known as polarographic GSH analysis can also be used to quantify GPx activity. Here, the GSH content is determined through polarography after stopping the GSH- H_2_O_2_ reactions at a specific time using a strong acid. However, this method has drawbacks, including sensitivity to oxygen, interference from other substances, and being time-consuming [95]. For the measurement of GPx concentration, ELISA is commonly used which allows for the detection of small amounts of GPx through a specific antigen-antibody reaction. However, this method lacks the ability to differentiate between functional and dysfunctional enzymes, resulting in the measurement of all GPx concentrations. Additionally, various companies offer commercially available that pose as convenient means to measure GPx activity [96].

1. Glutathione reductase (GR)

GR levels can be measured in serum and saliva using ELISA. For this, commercial ELISA kits are available [99, 100]. Various studies also measure the catalytic activity of GR in plasma. This is done by measuring the change in the absorbance (λ = 340 nm) caused due to the oxidation of NADPH to NADP+, in presence of GSSG. The rate of decrease in absorbance is proportional to the activity of glutathione reductase [101].

**Secondary Enzymes**

1. Thioredoxins (TRX)

TRX can be measured in serum and urine using ELISA [106, 107].

1. Thioredoxin peroxidases (PRX)

Monitoring oxidized PRX has proven to be valuable for measuring oxidative stress in erythrocytes. PRX2 is considerably high in erythrocytes, and exposure to low micromolar concentrations of H_2_O_2_ results in PRX2’s immediate hyperoxidation (PRX2-SO_2/3_) [111]. After PRX2 has reacted with H_2_O_2_, it takes 20–30 minutes for PRX2 to get converted back to its reduced form. This slow rate of reduction is attributed to the extremely low TRR activity in erythrocytes. The biological significance of this characteristic is unclear, but it helps in significantly increasing the sensitivity of PRX2 as a marker of oxidative stress in erythrocytes. Erythrocytic PRX2 can be assessed by observing hyperoxidized PRX2-SO_2/3_ levels in erythrocytes using Western blotting or mass spectrometry (Reverse Phase HPLC) [112].

1. Glutaredoxins (GRX)

A pilot study quantified GRX levels in serum using the Fluorescent GRX activity assay, using the method described by Coppo et al [114].

1. **Endogenous non-enzymatic antioxidants**
2. Glutathione (GSH)

The levels and activity of the isoforms, GSH and GSSG can be assessed in various biological samples, including whole blood, plasma, serum, tissues, and urine. Reduced GSH levels in whole blood are commonly used as markers of oxidative stress. GSH can be detected using Ellman's reagent followed by colorimetric or fluorometric quantification. Moreover, modern analytical techniques, such as LC-MS/MS, are employed to enable accurate GSH measurements [117]. Similarly, GSSG can be measured in blood, tissues, and urine, with blood GSSG levels often used to gauge the overall antioxidant capacity of an individual. Elevated GSSG levels in whole blood indicate the presence of oxidative stress. For precise and accurate measurement of GSSG, state-of-the-art analytical techniques like LC-MS/MS are employed [117]. Other methods of GSSG quantification include HPLC and spectrometry [118].

1. Uric Acid (UA)

UA can be measured in blood, serum, and urine. The two methods currently used to quantify UA are: colorimetric and enzymatic. The colorimetric method is based on the reduction of a chromogen such as sodium tungstate by UA to produce a measurable color change. This technique has been commonly employed in automated hospital screening (SMA systems). The enzymatic assay is based on the use of the enzyme uricase, which converts UA to allantoin and H_2_O_2_. Using this assay, UA can be measured directly and indirectly. The direct uricase assay quantifies the decrease of UV absorbance at 293 nm of UA upon uricase action while the indirect method quantifies the amount of H_2_O_2_ formed by uricase action. The direct method may have lower efficiency and precision while the indirect method may be sensitive to bilirubin, ascorbate, GSH, lipids, haemoglobin, and peroxide in serum besides xanthine [121]. Nevertheless, the uricase methods are currently available at comparable costs and are gradually replacing the less specific colorimetric method. Additionally, LC-MS-TOF, LC-MS/MS, and HPLC can be utilized as alternative methods to accurately quantify UA levels [122].

1. Bilirubin

Bilirubin can be measured in plasma, serum, urine, and feces. Among the different biological samples, serum samples are the most used sample for determining bilirubin. The diazo transfer reaction is the gold-standard assay for quantifying serum bilirubin [128]. However, alternative methods like HPLC, oxidative, enzymatic, and chemical methods, direct spectrophotometry, and transcutaneous methods are also used for bilirubin quantification. These alternative techniques have limitations in terms of sensitivity and specificity, and their accuracy can be affected by substances with color reactions. Another method involves chemiluminescence reaction using N-bromosuccinimide and sodium hypochlorite as oxidants, where bilirubin undergoes redox reactions in aqueous media, resulting in chemiluminescence. However, this technique lacks sufficient selectivity and sensitivity to various physiochemical factors. Polarographic approach and fluorometric approach are also utilized to measure bilirubin levels. While these methods offer higher sensitivity and the ability to detect lower quantities of the compound, they do have drawbacks. Polarography is sensitive to oxygen and may face interference from other substances, while fluorometry can be affected by sample matrix and background fluorescence [129].

1. Coenzyme Q10 (CoQ10)

CoQ10 can be measured in various biological samples, such as plasma, tissues, and platelets. It can be quantified as: total CoQ10, which represents the combined content of ubiquinol and ubiquinone, or the individual redox forms. Measuring total CoQ10 provides an overall representation of its antioxidant capacity, considering both ubiquinol and ubiquinone forms. In contrast, quantifying the individual redox forms provides valuable insights into their specific functions, aiding in understanding CoQ10's efficiency in neutralizing ROS. For accurate quantification of both total CoQ10 and individual redox forms, HPLC coupled with ECD, UV-detector, or mass spectrometer (HPLC-MS) emerges as the preferred choice. In modern analytical practices, cutting-edge techniques such as LC-MS/MS are employed to ensure precise and accurate measurements of CoQ10 levels [135].

III. **Exogenous Non-enzymatic Antioxidants**

1. Vitamin A

Vitamin A can be determined by evaluating the levels of retinoids and carotenoids in plasma, serum or tissue sample. This evaluation involves employing advanced analytical techniques, such as atmospheric pressure chemical ionization (APCI) liquid chromatography/mass spectrometry (LCMS) or reversed-phase high-performance liquid chromatography (HPLC) [140,141]. Through the application of these sophisticated methods, precise quantification of vitamin A and its derivatives is achieved, offering valuable insights into the antioxidant capacity and nutritional status of an individual. Additionally, this analysis helps to identify and address any deficiencies in the antioxidant vitamin A if present [141].

1. Vitamin C

Vitamin C can be measured in plasma, tissues, and urine with plasma samples being commonly assessed. Vitamin C can be quantified as total vitamin C concentration, which accounts for both, ascorbic acid and its oxidized form, DHA, or directly as ascorbic acid. EC-HPLC and UV-HPLC are widely recommended techniques for plasma total vitamin C analysis, as they can accurately quantify both forms. Alternatively, for direct quantification of ascorbic acid, spectrophotometry using the dinitrophenylhydrazine method and reversed-phase (RP) HPLC with UV or coulometric ECD is used [143]. It is worth noting that generally the estimates of intracellular ascorbic acid concentration vary due to its rapid degradation [144].

1. Vitamin E

Vitamin E can be measured to assess the body’s antioxidant capacity. It can be quantified in various biological samples including whole blood, plasma, serum and urine. Urinary vitamin E levels are frequently assessed using LC-MS/MS. However, its levels in serum can also be estimated using fluorometry. Alternatively, tocopherols can be analyzed by gas chromatography-mass spectrometry (GC-MS) on a SPB1 column using a selected ion monitoring technique or by reversed-phase HPLC [147].

1. Selenium

Serum and plasma samples are most tested for assessing selenium’s status in the body. Its concentrations in blood and urine are reflective of recent selenium intake. Currently, most clinical analyses for selenium continue to be performed using graphite furnace atomic absorption spectrometry, hydride-generation atomic absorption spectrometry (HGAAS) and molecular fluorescence spectrometry (MFS). Quantification of one or more selenoproteins (such as GPx, albumin, and selenoprotein P) is also used as a functional measure of selenium status. The concentrations of selenium associated with proteins in the serum can be quantified by using affinity HPLC coupled to inductively coupled plasma-mass spectrometry (ICP-MS). [149]

ANTIOXIDANT & OXIDANT SPECIES BALANCE

| **ACTION OF VARIOUS ANTIOXIDANTS IN THE BODY** | | | | |
| --- | --- | --- | --- | --- |
| **Category** | **Sub-category** | **Antioxidant** | **Oxidant species** | **Reference** |
| ***Enzymatic antioxidants*** | Primary enzymes | Superoxide dismutase (SOD) | Superoxide anion radical (O_2_^•^ˉ),  peroxynitrite (OONO^−^) | [85] |
|  |  | Catalase (CAT) | Hydrogen peroxide (H_2_O_2_),  Peroxynitrite (OONO^−^) | [85,  90] |
|  |  | Glutathione peroxidase (GPx) | Hydrogen peroxide (H_2_O_2_),  Lipid hydroperoxides (LOOHs) | [2, 85] |
|  | Secondary enzymes | Thioredoxin (TRX) | Hydrogen peroxide (H_2_O_2_), Hydroxyl radicals (^•^OH), peroxynitrite (OONO^−^) | [103] |
|  |  | Thioredoxin peroxidases (PRX) | Hydrogen peroxide (H_2_O_2_),  Alkyl hydroperoxides,  Peroxynitrite (OONO^−^) | [108] |
| ***Non-enzymatic Antioxidants*** | Endogenous | Glutathione (GSH) | Hydroxyl radicals (^•^OH),  Singlet Oxygen (^1^O_2_) | [115] |
|  |  | Uric acid (UA) | Peroxynitrite (OONO^−^)  Superoxide anion radical (O_2_^•^ˉ) | [120] |
|  |  | Albumin | Hydroxyl radicals (^•^OH) | [123] |
|  |  | Bilirubin | Peroxyl radicals (ROO^•^),  Singlet oxygen (^1^O_2_) | [127] |
|  |  | Coenzyme Q10 (CoQ10) | Hydrogen peroxide (H_2_O_2_), | [131] |
|  |  | Melatonin | Singlet oxygen (^1^O_2_), Superoxide anion radical (O_2_^•^ˉ),  Hydrogen peroxide (H_2_O_2_),  Nitric oxide (^•^NO),  Hypochlorous acid (HOCl) | [136] |
|  |  | Alpha-Lipoic acid (ALA) | Superoxide anion radical (O_2_^•^ˉ),  Hydroxyl radicals (^•^OH),  Hypochlorous acid (HOCl) | [137,  138] |
|  | Exogenous | Vitamin A | Singlet oxygen (^1^O_2_),  Lipid hydroperoxides (LOOHs) | [139] |
|  |  | Vitamin C | Hydroxyl radicals (^•^OH), Hydrogen peroxide (H_2_O_2_),  Singlet oxygen (^1^O_2_) | [142] |
|  |  | Vitamin E | Superoxide anion radical (O_2_^•^ˉ),  Peroxyl radicals (ROO^•^) | [145] |
|  |  | Selenium | Hydrogen peroxide (H_2_O_2_) | [148] |
|  |  | Polyphenols | Nitric oxide (^•^NO) | [151] |

Table 1. Action of various antioxidants in the body
